# Steroid hormone bioavailability is controlled by the lymphatic system

**DOI:** 10.1038/s41598-021-88508-w

**Published:** 2021-05-06

**Authors:** Rahel Klossner, Michael Groessl, Nadine Schumacher, Michaela Fux, Geneviève Escher, Sophia Verouti, Heidi Jamin, Bruno Vogt, Markus G. Mohaupt, Carine Gennari-Moser

**Affiliations:** 1 Department of Nephrology and Hypertension, University of Bern, 3010 Bern, Switzerland; 2 Department of Medicine, Lindenhofgruppe, 3006 Bern, Switzerland; 3 Department for BioMedical Research, University of Bern, 3010 Bern, Switzerland; 4 Department for Clinical Chemistry, Inselspital, 3010 Bern, Switzerland; 5 Campus SLB, Sitem, 3010 Bern, Switzerland; 6 Division of Child Health, Obstetrics and Gynecology, University of Nottingham, Nottingham, NG5 1PB UK

**Keywords:** Cancer, Endocrinology

## Abstract

The steroid hormone progesterone accounts for immune tolerance in pregnancy. Enhanced progesterone metabolism to 6α-OH-pregnanolone occurs in complicated pregnancies such as in preeclampsia with preterm delivery or intrauterine growth restriction, and in cancer. As lymphatic endothelial cells (LECs) promote tumor immunity, we hypothesized that human LECs modify progesterone bioavailability. Primary human LECs and mice lymph nodes were incubated with progesterone and progesterone metabolism was analyzed by thin layer chromatography and liquid chromatography-mass spectrometry. Expression of steroidogenic enzymes, down-stream signal and steroid hormone receptors was assessed by Real-time PCR. The placental cell line HTR-8/SV neo was used as reference. The impact of the progesterone metabolites of interest was investigated on the immune system by fluorescence-activated cell sorting analysis. LECs metabolize progesterone to 6α-OH-pregnanolone and reactivate progesterone from a precursor. LECs highly express 17β-hydroxysteroid dehydrogenase 2 and are therefore antiandrogenic and antiestrogenic. LECs express several steroid hormone receptors and PIBF1. Progesterone and its metabolites reduced TNF-α and IFN-γ production in CD4+ and CD8+ T cells. LECs modify progesterone bioavailability and are a target of steroid hormones. Given the global area represented by LECs, they might have a critical immunomodulatory control in pregnancy and cancer.

## Introduction

The lymphatic system, especially lymphatic endothelial cells (LECs) are thought to play a crucial role in tumor immunity. For example, tumorigenic cells travelling inside lymphatic vessels towards sentinel lymph nodes are not being recognized as foreign and an appropriate immune response is missing, leading to tumor survival^1^. Additionally, the expression and release of the lymphangiogenic factor VEGF-C by tumors is linked to metastasis, poor prognosis and immune tolerance^2^. LECs are, like dendritic cells, antigen-presenting cells that mediate systemic peripheral immune tolerance^3,4^. Upon presentation of self-antigens by LECs, T-cells undergo apoptosis and LECs therefore contribute to immune tolerance. LECs also promote immune tolerance when tumorigenic antigens are presented to T-cells. T-cells, which were activated by LECs, become more rapidly apoptotic as T-cells activated by mature dendritic cells^5^. Though preventing autoimmune reactions and promoting immune tolerance during pregnancy, this behavior may favor tumor development. LECs in the local microenvironment of a tumor might therefore be a target for immunomodulation^5^.

During pregnancy, immune tolerance is required and linked to high progesterone levels^6,7^. This immunomodulatory effect of progesterone is mediated via upregulation of the lymphocyte-derived progesterone-induced blocking factor (PIBF)^8–13^. Thus, in pregnant women^14^ and cancer patients^15^, there is an increased progesterone metabolism.

The first step in progesterone metabolism leads to the formation of active 5α-dihydroprogesterone (5α-DHP), which is further metabolized into the 6α-OH-pregnanolones (5α-pregnan-3α/β,6α-diol-20-one) and allopregnane-3,20-diol (5α-pregnan-3β,20α-diol). The metabolism of 5α-DHP is extrahepatic, as the final step of 6α-OH-pregnanolones and allopregnane-3,20-diol formation is not catalyzed by the cytochrome P450 steroid 6α-hydroxylase expressed in the liver^16^. Horwitz et al. proposed an intracellular metabolism of progesterone into the 6α-OH-pregnanolones involving 3 different enzymes: 5α-reductase (SRD5A1-3), 3β-hydroxysteroid dehydrogenases (HSD3B1/2), and a 6α-hydroxylase^17^.

Several reports have demonstrated that the 5α- and 5β-DHP metabolites are biologically active in pregnancy^18–22^, but the mechanism leading to these metabolites remains to be elucidated. Allopregnanolone, the metabolite down-stream of 5α-DHP, stimulates proliferation and migration in the ovarian cancer cell line IGROV-1^15^ and in glioblastoma^23^.

Several enzymes accounting for steroid hormone metabolism, specifically progesterone metabolism, have been involved in cancer. These are the 6α-hydroxylase, the 5α-reductase, the 3β-hydroxysteroid dehydrogenase and 17β-hydroxysteroid dehydrogenase 2 (HSD17B2)^24–29^. The progesterone metabolite 5α-DHP produced by the enzyme 5α-reductase, is a target for prostate cancer and glioblastoma therapy^30–33^. Interestingly, HSD17B2 also exerts a high 20α-hydroxysteroid dehydrogenase activity^34^ and can convert the weak 20α-hydroxyprogesterone (20α-OHP) into progesterone^35–38^. Many compounds targeting HSD17B2 activity in various cancers are under investigation^39^.

The hypothesis of this investigation is that human LECs modify the bioavailability of progesterone. To prove this, **first,** progesterone metabolism was assessed in primary human LECs isolated from lymph nodes (HLEC) and results were confirmed in LECs isolated from lymphatic skin vessels (dLEC); **second,** the resulting progesterone metabolite was identified and characterized by LC–MS; **third,** the enzymatic steps involved were clarified; **fourth,** human cell culture results were matched to *ex-vivo* mouse lymph node; **fifth,** the impact of the found progesterone metabolites 5α-dihydroprogesterone (5α-DHP) and 6α-OH-pregnanolone (6α-OH-Pregn) was tested on immune cells. The placental cell line HTR-8/SVneo was used as a control and reference for the experiments with lymphatic endothelial cells.

## Results

### Production of de-novo steroid hormones from cholesterol in HLEC and dLEC

HLECs and dLECs were cultured in steroid-free medium and incubated with ^3^H-cholesterol to quantify the *de-novo* synthesis of progesterone, corticosterone, cortisol, testosterone and estradiol (HLEC), and of progesterone, 11-deoxycortisol, corticosterone, aldosterone and cortisol (dLEC), after an incubation time of 24 h. No significant *de-novo* steroid hormone production could be detected out of the precursor cholesterol by thin layer chromatography (TLC) in HLEC (Supplementary Fig. 1a online) and dLEC (Supplementary Fig. 1b online).

**Figure 1:**
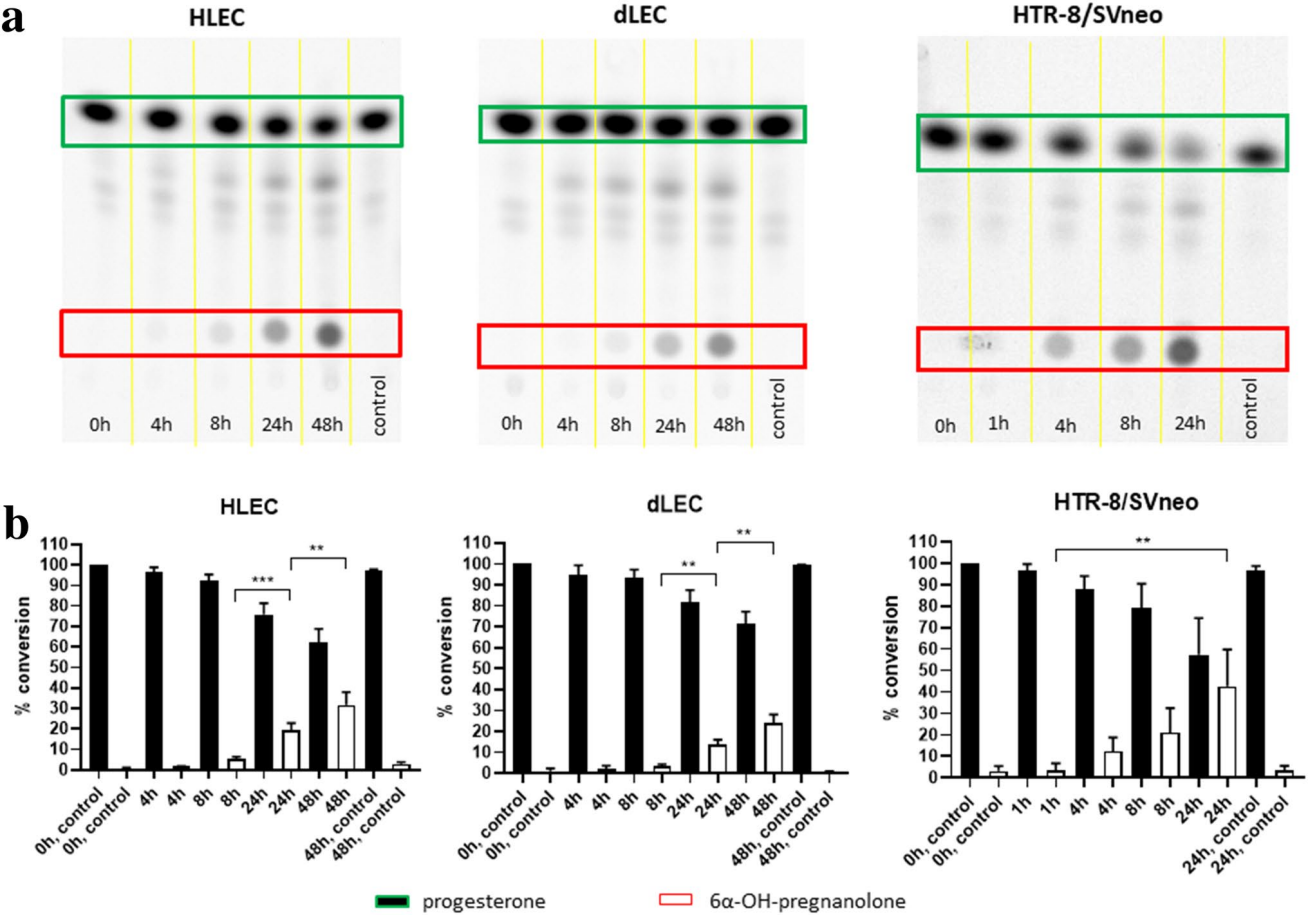
**Characterization of progesterone metabolism in HLEC, dLEC and HTR8/SV neo by TLC**. Metabolism of progesterone in HLEC, dLEC and HTR-8/SV neo. (**a**) Characteristic phosphorimager pictures of time-dependent conversion of progesterone to a main metabolite, 6α-OH-pregnanolone. Cell-free controls were run for time point 0 h (HLEC, dLEC and HTR-8/SV neo), for time point 24 h (HTR-8/SVneo) and for time point 48 h (HLEC and dLEC). (**b**) Densitometric quantification of all performed experiments. Progesterone was time-dependently and significantly converted to 6α-OH-pregnanolone in all three cell lines. Data were normalized to condition 0 h = 100% progesterone. One-way ANOVA, Dunnett’s multiple comparisons test, n = 5. Production of 6α-OH-pregnanolone: HLEC: 8 h-24 h ***p < 0.0001, 24 h-48 h **p = 0.0002. dLEC: 8 h-24 h ** *p* = 0.0026, 24 h-48 h ** *p* = 0.0020. HTR-8/SV neo: 1 h-24 h ** *p* = 0.0002. Green rectangle/black column: substrate (progesterone). Red rectangle/white column: product (6α-OH-pregnanolone). * *p* < 0.05, ** *p* < 0.01, *** *p* < 0.0001, ns = not significant.

### Characterisation of progesterone metabolism in HLEC, dLEC and HTR-8/SVneo by TLC

HLECs, dLECs and HTR-8/SVneo cells were cultured in steroid-free medium and incubated with ^14^C-progesterone for 4 h/8 h/24 h/48 h (HLEC/dLEC) or 1 h/4 h/8 h/24 h (HTR-8/SVneo). Steroids were extracted from supernatants and the metabolites separated by TLC. Progesterone was time-dependently and significantly converted to one major steroid hormone, 6α-OH-pregnanolone, in all three cell lines with a production rate of 19.3% ± 3.6 (24 h) and 31.7% ± 6.3 (48 h) in HLEC, 13.6% ± 2.5 (24 h) and 23.9% ± 4.2 (48 h) in dLEC and 21.0% ± 11.5 (8 h) and 42.7% ± 17.1 (24 h) in HTR-8/SV neo (Fig. 1). One-way ANOVA, Dunnett’s multiple comparisons test. Mean ± SD, n = 5.

### Characterization of 6α-OH-pregnanolone in HLEC, dLEC and HTR-8/SVneo by LC–MS

**Figure 2:**
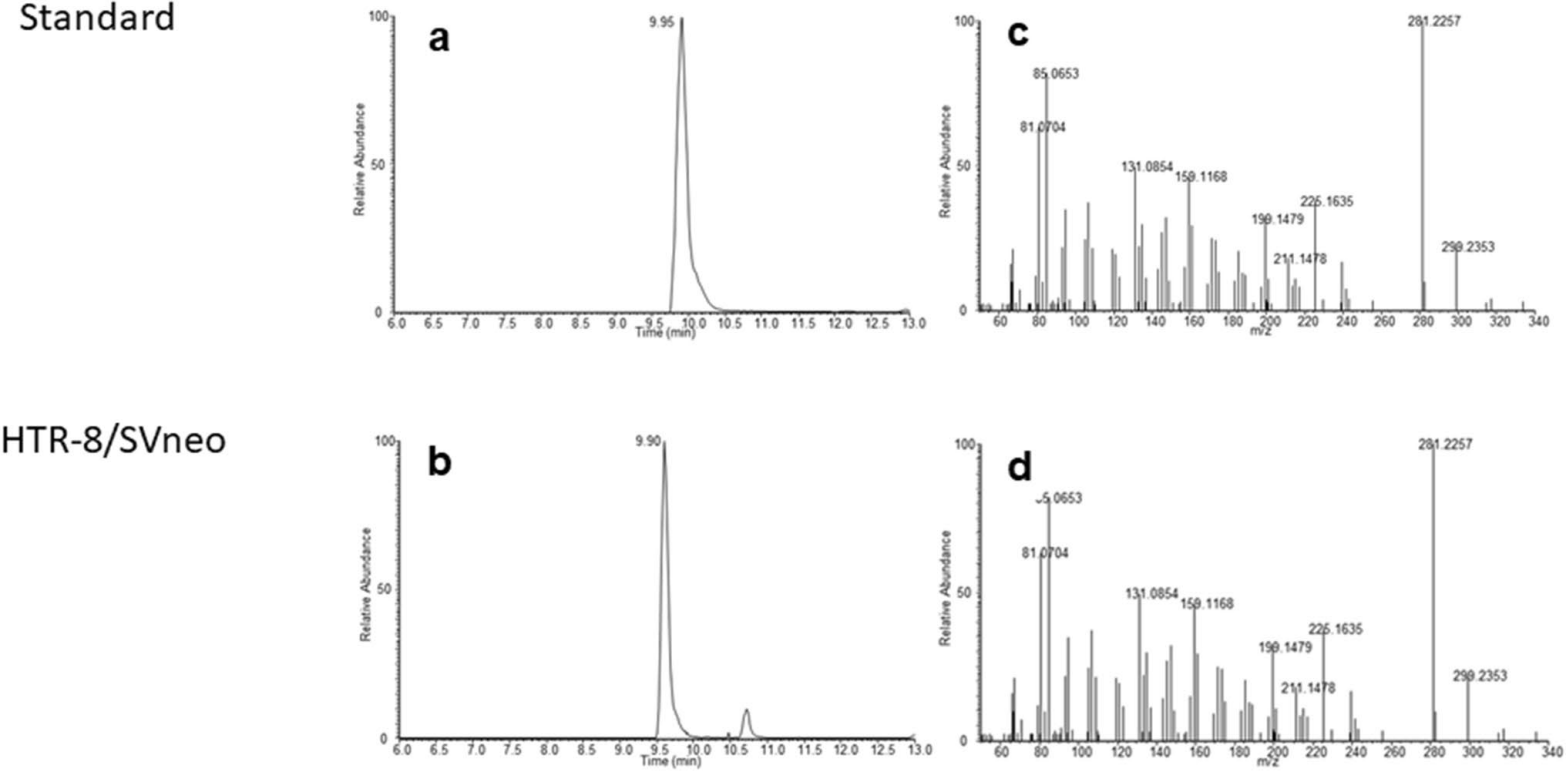
Identification of 6α-OH-pregnanolone by LC–MS/MS in HTR-8/SV neo cells: LC–MS trace of m/z 317.2469 corresponding to a sum formula of C_21_H_34_O_3_ in an authentic standard (**a**) and in HTR-8/SV neo cells (**b**). MS/MS fragment spectra recorded at LC–MS peak maximum for an authentic standard (**c**) and in HTR-8/SVneo cells (**d**).

**Figure 3:**
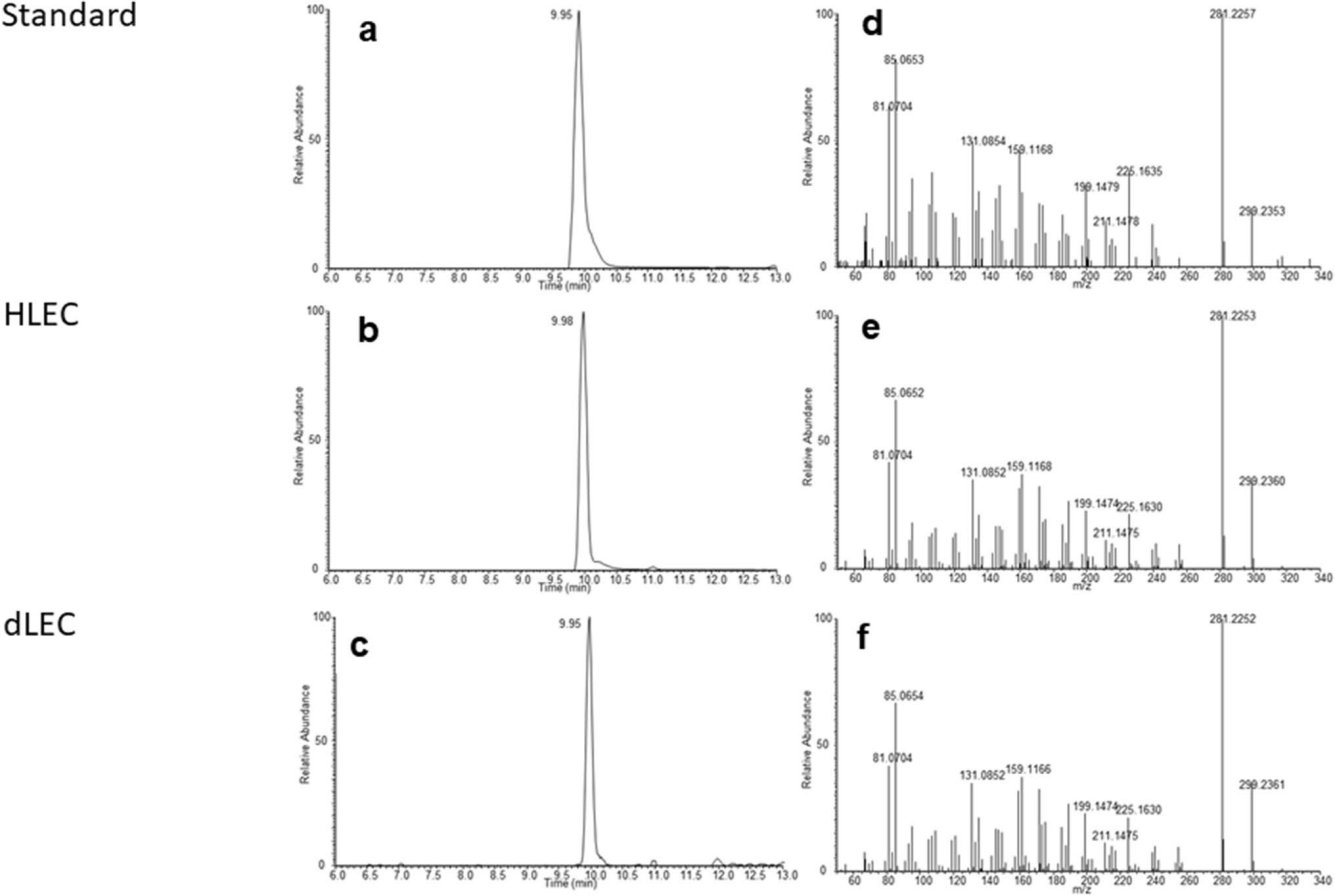
Identification of 6α-OH-pregnanolone by LC–MS/MS in HLEC and dLEC cells. LC–MS trace of m/z 317.2469 corresponding to a sum formula of C_21_H_34_O_3_ in an authentic standard (**a**), in HLEC cells (**b**) and in dLEC cells (**c**). MS/MS fragment spectra recorded at LC–MS peak maximum in an authentic standard (**d**), in HLEC cells (**e**) and in dLEC cells (**f**).

In order to validate the results obtained by TLC, steroidomic analysis of cell supernatants was performed using LC–MS. Samples were measured in untargeted mode and investigated for peaks that showed strongest signal increase over the time course experiment. Subsequently, the accurate mass of peaks of interest was compared with the theoretical value for 6α-OH-pregnanolone and both, fragmentation spectrum and retention time, were compared to an authentic standard (Figs. 2 and 3). The LC–MS data clearly confirms the production of 6α-OH-pregnanolone from progesterone as obtained by TLC.

### Gene expression analysis in HLEC, dLEC and HTR-8/SVneo

HLECs, dLECs and HTR-8/SV neo cells were cultured as described in methods. RNA extraction and Real-time PCR was conducted using assay on demand primers or primers and probes from the Roche library (Tables 1, 2). Cyclophilin A served as endogenous control.

**Table 1:** Human primers and probes used in Real-time PCR.

Gene	Accession number	Primers	Probe	Amplicon size (nt)
SRD5A1	NM_001324322.1	5′-ttg gag aaa tca tgg agt ggt-3’	37	141
		5′-act ctt caa att tcc gga ggt a-3’		
SRD5A2	NM_000348.3	5′-cag cta cag gat tcc aca agg-3’	50	72
		5′-tca atg atc tca ccg agg aa-3’		
SRD5A3	NM_024592.4	5′-ggc ttc atg gtt tgc tca g-3’	24	96
		5′-gca gcc aca gaa ata cta gca c-3’		
AKR1D1	NM_001190906.1	5′-cca aaa tga aca cga agt tgg-3’	7	108
		5′-aca tga ttt gta gcc cat agc tt-3’		
AKR1C1	NM_001353.5	5′-cat gcc tgt cct ggg att t-3’	49	109
		5′-aga atc aat atg gcg gaa gc-3’		
AKR1C2	NM_001354.5	5′-cta tgc gcc tgc aga ggt-3’	31	114
		5′-acc tgc tcc tca tta ttg taa aca t-3’		
AKR1C3	NM_003739.5	5′-cat tgg ggt gtc aaa ctt ca-3’	27	112
		5′-ccg gtt gaa ata cgg atg ac-3’		
AKR1C4	NM_001818.3	5′-agg tga gac gcc act acc aa-3’	53	96
		5′-tcc tta cac ttc tcc atg acc tc-3’		
HSD3B1	NM_000862.2	5′-atc atc cgc ctc ttg gtg-3’	17	114
		5′-cag ctt ggt ctt gtt ctg ga-3’		
HSD3B2	NM_000198.3	5′-ctt gga caa ggc ctt cag ac-3’	50	78
		5′-tca agt aca gtc agc ttg gtc ct-3’		
HSD17B2	NM_002153.2	5′-gcc aag aat tgt tac ctg tgg-3’	8	88
		5′-tcc aga tac ttg cac aaa gca-3’		
HSD11B1	NM_001206741.1	5′-tct gtg ttc ttg gcc tca tag a-3'	8	75
		5′-gag ctg ctt gca tat gga cta tc-3'		
FDXR	NM_001258015.2	5′-tcc tac tga ccc cac ctg ag-3'	8	77
		5′-tcg ac tct gcc tca gta cac c-3'		
NR5A2	NM_205860.2	5′-ccg aca agt ggt aca tgg aa-3'	61	88
		5′-tcc ggc ttg tga tgc tat ta-3'

**Table 2:** Human assay on demand primers (Applied Biosystems) used in Real-time PCR.

Gene	# Cat Nr
PIBF1	Hs00197131_m1
NR3C1	Hs00353740_m1
NR3C2	Hs01031809_m1
NR3C3	Hs01556702_m1
PGRMC1	Hs009998344_m1
PGRMC2	Hs01128672_m1
GPER1	Hs00173506_m1
CYP17A1	Hs01124136_m1
CYP21A2	Hs00416901_g1
CYP11B1	Hs01596404_m1
CYP11B2	Hs01597732_m1
HSD11B2	Hs00388669_m1
StAR	Hs00986559_g1
FDX1	Hs01070067_g1

The mRNA expression of enzymes involved in progesterone metabolism (Table 3) was quantified first. HLEC and dLEC express high levels of SRD5A1, SRD5A3, AKR1C1, AKR1C2, AKR1C3, but they do not express SRD5A2, AKR1D1, and AKR1C4. No expression of HSD3B1/2 was found (Ct > 35). HTR-8/SV neo express high levels of SRD5A1, SRD5A3, AKR1C2 and AKR1C3, but rather low levels of SRD5A2 and AKR1C1. AKR1D1 and AKR1C4 as well as HSD3B1/2 are not expressed in HTR-8/SV neo (Ct > 35).

**Table 3:**
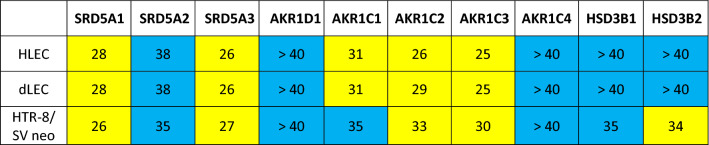
Gene expression analysis in HLEC, dLEC and HTR-8/SVneo. mRNA expression of enzymes involved in progesterone metabolism in HLEC, dLEC and HTR-8/SV neo. Expression levels are shown as ct values per 12.5 ng cDNA.

Yellow: significant expression (ct < 35).

Blue: no or very low expression (ct ≥ 35).

SRD5A1 (5α-reductase isoform 1), SRD5A2 (5α-reductase isoform 2), SRD5A3 (5α-reductase isoform 3), AKR1D1 (5β-reductase), AKR1C1 (20α-HSD), AKR1C2 (3α-HSD3), AKR1C3 (HSD17B5), AKR1C4 (3α-HSD1), HSD3B1, HSD3B2. Appropriate positive and negative controls were run.

Next, the mRNA expression of the progesterone regulated gene (PIBF1) and of steroid hormone receptors in HLEC, dLEC and HTR-8/SV neo was assessed (Table 4). All three cell lines significantly express PIBF1 as well as the glucocorticoid (NR3C1) and the mineralocorticoid receptor (NR3C2), the two membrane bound progesterone receptors type 1 and 2 (PGRMC1 and 2) and the estrogen receptor (GPER1). They show no or very low expression of the nuclear progesterone receptor (NR3C3).

**Table 4:**
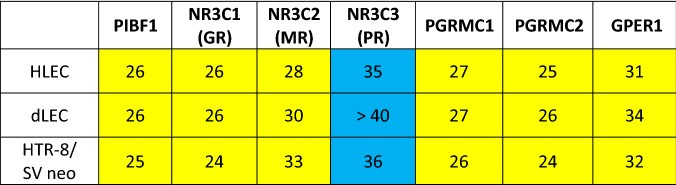
Gene expression analysis in HLEC, dLEC and HTR-8/SVneo. mRNA expression of PIBF1, and of steroid hormone receptors in HLEC, dLEC and HTR-8/SV neo. Expression levels are shown as ct values per 12.5 ng cDNA.

Yellow: significant expression (ct < 35).

Blue: no or very low expression (ct ≥ 35).

PIBF1 (progesterone induced blocking factor 1), NR3C1 (glucocorticoid receptor), NR3C2 (mineralocorticoid receptor), NR3C3 (progesterone receptor), PGRMC1 (progesterone receptor membrane component 1), PGRMC2 (progesterone receptor membrane component 2), GPER1 (G protein-coupled estrogen receptor 1). Appropriate positive and negative controls were run.

Steroidogenic enzymes involved in de-novo steroid hormone production were assessed thereafter (Table 5). HLEC and dLEC express no CYP17A1, CYP21A2, CYP11B1, CYP11B2, HSD11B1 and no HSD11B2, but they highly express HSD17B2. HTR-8/SV neo express no CYP17A1, CYP21A2, CYP11B1, CYP11B2, and no HSD11B1 but they express HSD11B2 and HSD17B2.

**Table 5:**
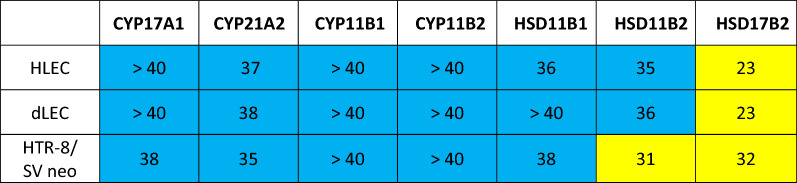
Gene expression analysis in HLEC, dLEC and HTR-8/SVneo. mRNA expression of steroidogenic enzymes involved in de-novo steroid hormone production in HLEC, dLEC and HTR-8/SV neo. Expression levels are shown as ct values per 12.5 ng cDNA. Yellow: significant expression (ct < 35). Blue: no or very low expression (ct ≥ 35). CYP17A1 (steroid-17α-hydroxylase), CYP21A2 (steroid-21-hydroxylase), CYP11B1 (steroid-11β-hydroxylase), CYP11B2 (aldosterone synthase), HSD11B1 (11β-hydroxysteroid-dehydrogenase 1), HSD11B2 (11β-hydroxysteroid-dehydrogenase 2), HSD17B2 (17β-hydroxysteroid dehydrogenase 2). Appropriate positive and negative controls were run.

Assessment of the mRNA expression of steroidogenic proteins involved in de-novo steroid hormone production (Table 6) revealed no significant expression of StAR in HLEC and dLEC, while FDXR, FDX1 and NR5A2 were strongly expressed. HTR-8/SV neo significantly expressed StAR, FDXR, FDX1 and NR5A2.

**Table 6:**
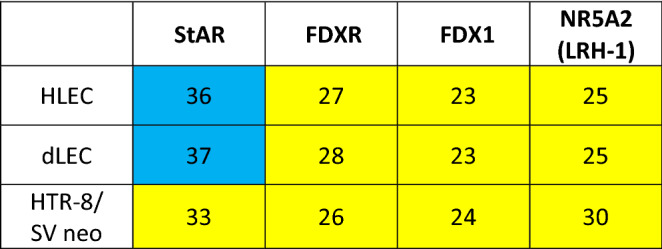
Gene expression analysis in HLEC, dLEC and HTR-8/SVneo. mRNA expression of steroidogenic proteins involved in de-novo steroid hormone production in HLEC, dLEC and HTR-8/SV neo. Expression levels are shown as ct values per 12.5 ng cDNA.

Yellow: significant expression (ct < 35).

Blue: no or very low expression (ct ≥ 35).

CYP17A1 (steroid-17α-hydroxylase), CYP21A2 (steroid-21-hydroxylase), CYP11B1 (steroid-11β-hydroxylase), CYP11B2 (aldosterone synthase), HSD11B1 (11β-hydroxysteroid-dehydrogenase 1), HSD11B2 (11β-hydroxysteroid-dehydrogenase 2), HSD17B2 (17β-hydroxysteroid dehydrogenase 2). Appropriate positive and negative controls were run.

### Derived cascade of progesterone metabolism in HLEC, dLEC and HTR-8/SV neo

**Figure 4:**
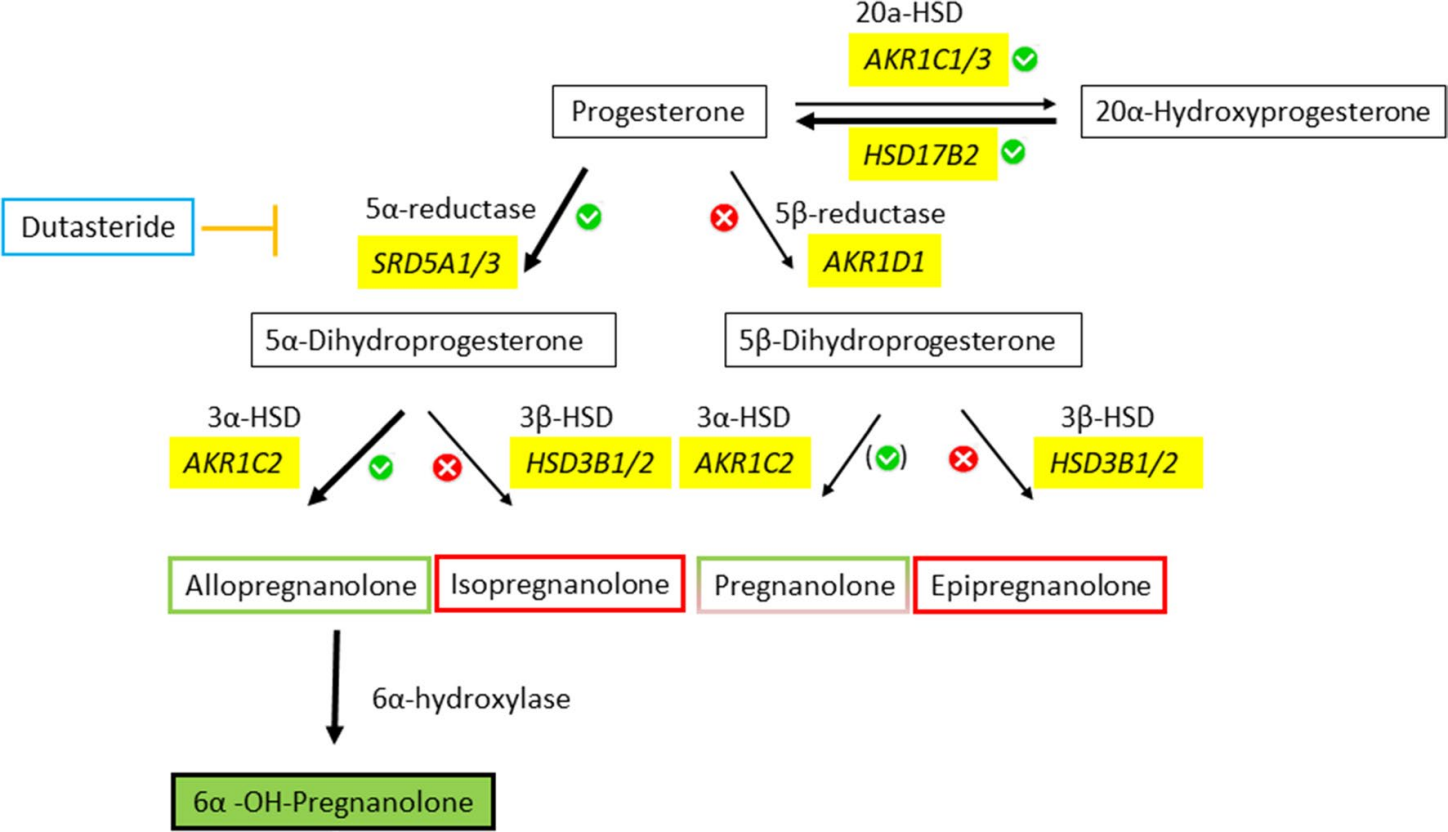
Putative progesterone metabolism pathway in HLECs, dLECs and HTR-8/SV neo. Based on our mRNA, proteomics, TLC and LC–MS data, we propose the following pathway in green to take place. The pathway with the bulky arrows down to 6α-OH-pregnanolone is favored over the pathway to the 20α-hydroxyprogesterone in HLECs, dLECs and HTR-8/SV neo. HLEC and dLEC highly express HSD17B2 and efficiently back-convert 20α-hydroxyprogesterone to progesterone.

Based on our findings by Real-time PCR, LC–MS and Thin layer chromatography, progesterone is metabolized via 5α-DHP and allopregnanolone to 6α-OH-pregnanolone in HLECs, dLECs and HTR-8/SVneo (Fig. 4). Furthermore, HLEC and dLEC efficiently back-convert 20α-hydroxyprogesterone to progesterone due to high HSD17B2 expression.

### Inhibition of 6α-OH-pregnanolone formation in HLEC, dLEC and HTR-8/SV neo using the SRD5A1-3 inhibitor dutasteride

**Figure 5:**
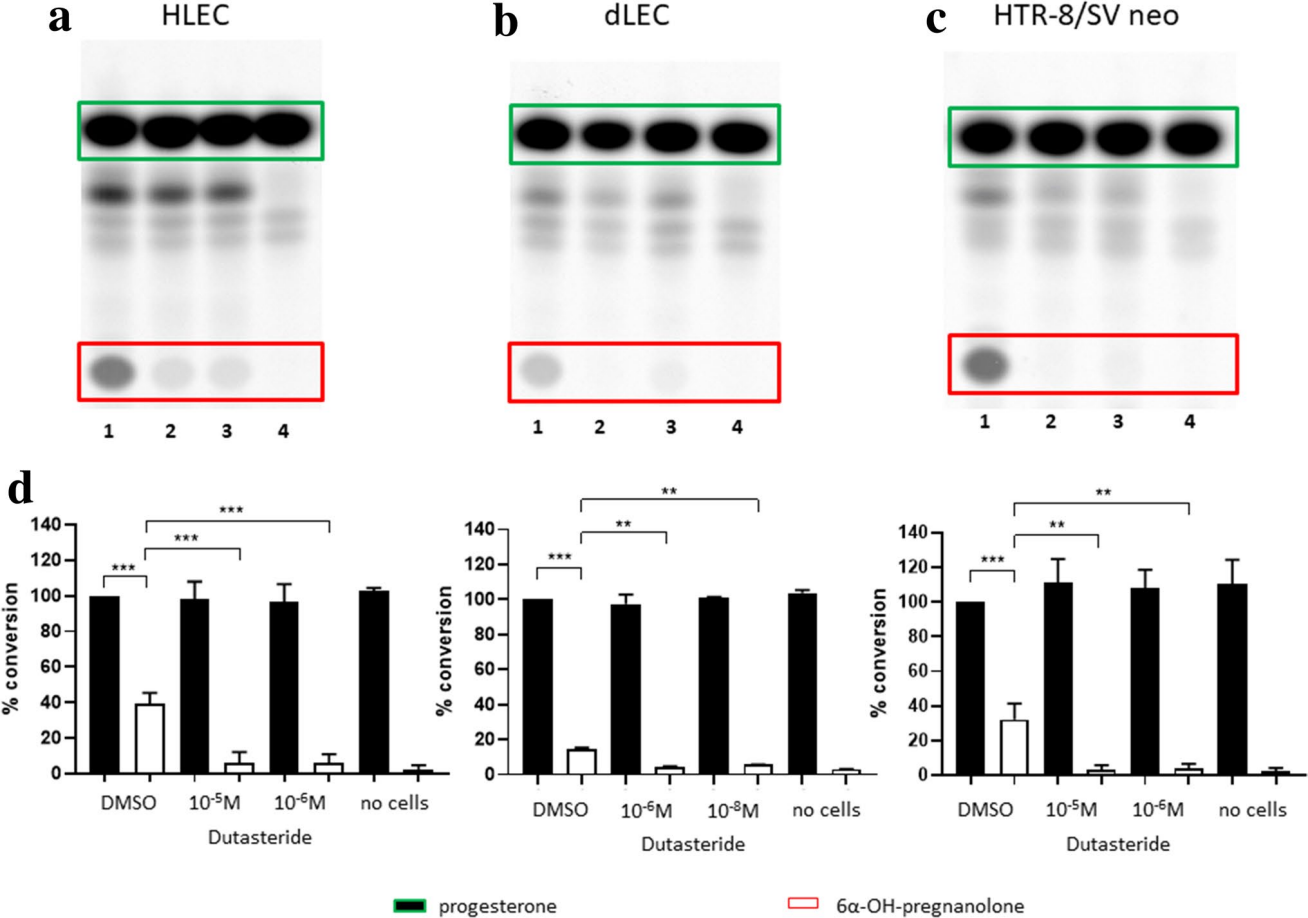
Inhibitory effect of dutasteride on 5α-reductase (SRD5A1-3) in HLECs, dLECs and HTR-8/SV neo. Characteristic phorphorimager pictures of (**a**) HLEC, (**b**) dLEC, (**c**) HTR-8/SV neo and their densitometry (**d**) of three independent experiments. Cells were cultured without (lane 1) and with (lane 2 and 3) the indicated concentration of dutasteride and 14C-progesterone. Lane 4 is the cell-free control, where 14C-progesterone was incubated for 24 h without cells. HLEC and HTR-8/SV neo: (1) no dutasteride = DMSO control, (2) dutasteride 10−5 M, (3) dutasteride 10−6 M, (4) no cells; dLEC: (1) no dutasteride, (2) dutasteride 10−6 M, (3) dutasteride 10−8 M, (4) no cells. Progesterone was significantly metabolized to 6α-OH-pregnanolone in the DMSO control, while dutasteride significantly inhibited the conversion of progesterone to 6α-OH-pregnanolone in all three cell lines. Data were normalized to condition 0 h = 100% progesterone. One-way ANOVA, Dunnett’s multiple comparisons test, n = 3. HLEC: DMSO control *** p < 0.0001; dutasteride 10−5 M *** p < 0.0001; dutasteride 10−6 M *** p < 0.0001. dLEC: DMSO control *** p < 0.0001; dutasteride 10−6 M ** p = 0.0001; dutasteride 10−8 M ** p = 0.0006. HTR-8/SV neo: DMSO control *** p < 0.0001; dutasteride 10−5 M ** p = 0.0005; dutasteride 10−6 M ** p = 0.0006. Green rectangle/black column: substrate (progesterone). Red rectangle/white column: product (6α-OH-pregnanolone). * p < 0.05, ** p < 0.01, *** p < 0.0001, ns = not significant.

HLECs, dLECs and HTR-8/SV neo cells were cultured and incubated for 24 h with the 5α-reductase inhibitor dutasteride at 2 different concentrations, 10^−5^ M and 10^−6^ M for HLEC and HTR-8/SV neo and 10^−6^ M and 10^−8^ M for dLEC. Supernatants were analyzed by TLC to measure the conversion of ^14^C-progesterone to 6α-OH-pregnanolone (Fig. 5). Dutasteride significantly inhibited 6α-OH-pregnanolone formation at both concentrations in all three cell lines. In untreated HLECs (DMSO) 39.2% ± 6.3% 6α-OH-pregnanolone was produced from progesterone (Fig. 5a). This production was reduced to 6.3% ± 5.8%, respectively 6.2% ± 4.7% with dutasteride 10−5 M and 10−6 M. A similar inhibitory effect by dutasteride was observed in dLECs (Fig. 5b) and HTR-8/SV neo cells (Fig. 5c). In dLECs, production of 6α-OH-pregnanolone went from 14.5% ± 0.9% to 4.1% ± 0.6% with dutasteride 10−6 M and to 5.7% ± 0.2% with dutasteride 10−8 M. Similarly, HTR-8/SV neo cells produced 32.1% ± 9.1% 6α-OH-pregnanolone (DMSO), and this was reduced to 3.0% ± 2.7% with dutasteride 10−5 M and to 3.7% ± 2.9% with dutasteride 10−6 M. The involvement of the SRD5A1-3 for the production of 6α-OH-pregnanolone was confirmed by enzymatic activity and RT-PCR. One-way ANOVA, Dunnett’s multiple comparisons test. Mean ± SD, n = 3.

### Detection of HSD17B2 protein and its activity in HLEC, dLEC and HTR-8/SV neo

**Figure 6:**
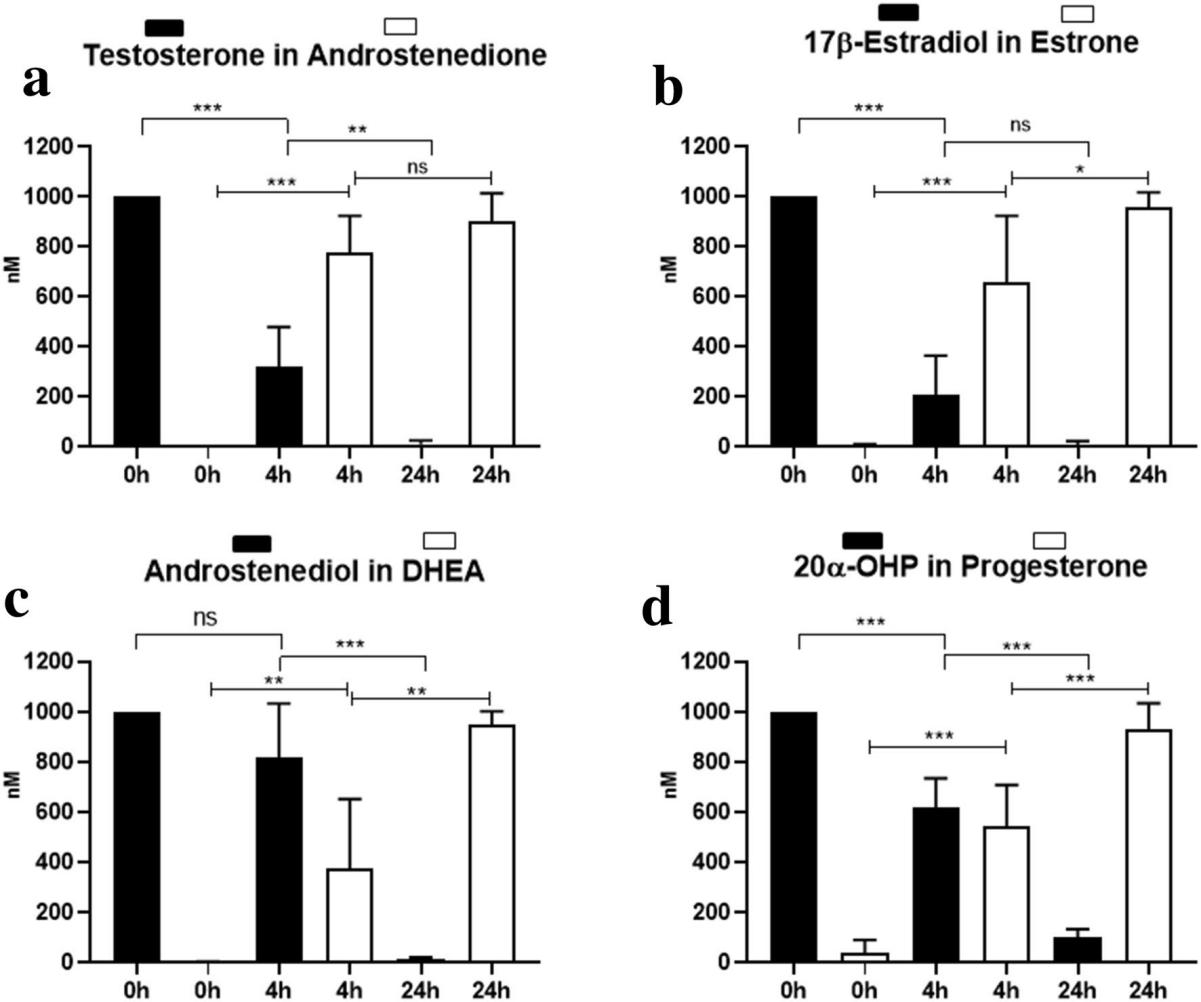
Activity of HSD17B2 in HLECs. Time-dependent conversion of testosterone to androstenedione, 17β-estradiol to estrone, androstenediol to DHEA-S and DHEA as well as 20α-hydroxyprogesterone to progesterone was assessed by LC–MS. Data were normalized to condition at 0 h = 1000 nM = 10−6 M for each compound. Y-axis shows steroid hormone concentrations in nM. Testosterone was significantly converted to androstenedione (testosterone 0 h/4 h/24 h: *** p < 0.0001/** p = 0.0019; androstenedione 0 h/4 h/24 h: *** p < 0.0001/ns). 17β-estradiol was significantly converted to estrone (17β-estradiol 0 h/4 h/24 h: *** p < 0.0001/ns; estrone 0 h/4 h/24 h: *** p < 0.0001/* p = 0.01). Androstenediol was significantly converted to DHEA (androstenediol 0 h/4 h/24 h: ns/*** p < 0.0001; DHEA 0 h/4 h/24 h: ** p = 0.0087/** p = 0.0002). 20α-OHP was significantly converted to progesterone (20α-OHP 0 h/4 h/24 h: *** p < 0.0001/*** p < 0.0001; progesterone 0 h/4 h/24 h: *** p < 0.0001/*** p < 0.0001). One-way ANOVA, Dunnett’s multiple comparisons test, n = 3. Black rectangle/column: substrate. White rectangle/column: product. * p < 0.05, ** p < 0.01, *** p < 0.0001, ns = not significant.

Proteomics analysis revealed a strong expression of HSD17B2 protein in HLEC and dLEC. HTR-8/SV neo also expressed HSD17B2, but much weaker than the LECs. HSD17B2 activity was next quantified by LC–MS by measuring the conversion of testosterone to androstenedione, of 17β-estradiol to estrone, of androstenediol to dehydroepiandrosterone **(**DHEA) and DHEA-S and of 20α-OHP to progesterone. There was a significant HSD17B2 activity, both in HLEC (Fig. 6) and dLEC (data not shown). LECs, completely converted testosterone, 17β-estradiol, androstenediol and 20α-OHP to androstenedione, estrone, DHEA/DHEA-S and progesterone within 24 h. In HLECs, testosterone (0 h: 1000 ± 0; 4 h: 318.7 ± 160.6, 24 h: 10.3 ± 14.9) was time-dependently converted to androstenedione (0 h: 1.4 ± 1.0; 4 h: 776.6 ± 148.1; 24 h: 899.9 ± 114.4) (Fig. 6a); 17β-estradiol (0 h: 1000 ± 0; 4 h: 205.1 ± 159.7; 24 h: 7.7 ± 15.3) to estrone (0 h: 6.5 ± 4.4; 4 h: 659.4 ± 263.9; 24 h: 955.5 ± 62.0) (Fig. 6b); Androstenediol (0 h: 1000 ± 0; 4 h: 816.5 ± 217.2; 24 h: 14.3 ± 6.5) to DHEA (0 h: 2.2 ± 2.3; 4 h: 375.0 ± 277.6; 24 h: 946.3 ± 55.8) (Fig. 6c) and 20α-OHP (0 h: 1000 ± 0; 4 h: 618.9 ± 116.6; 24 h: 101.9 ± 31.9) to progesterone (0 h: 39.0 ± 51.2; 4 h: 542.3 ± 166.6; 24 h: 929.6 ± 104.6) (Fig. 6d). One-way ANOVA, Dunnett’s multiple comparisons test. Mean ± SD, n = 3.

### Progesterone metabolism in mouse lymph nodes

**Figure 7:**
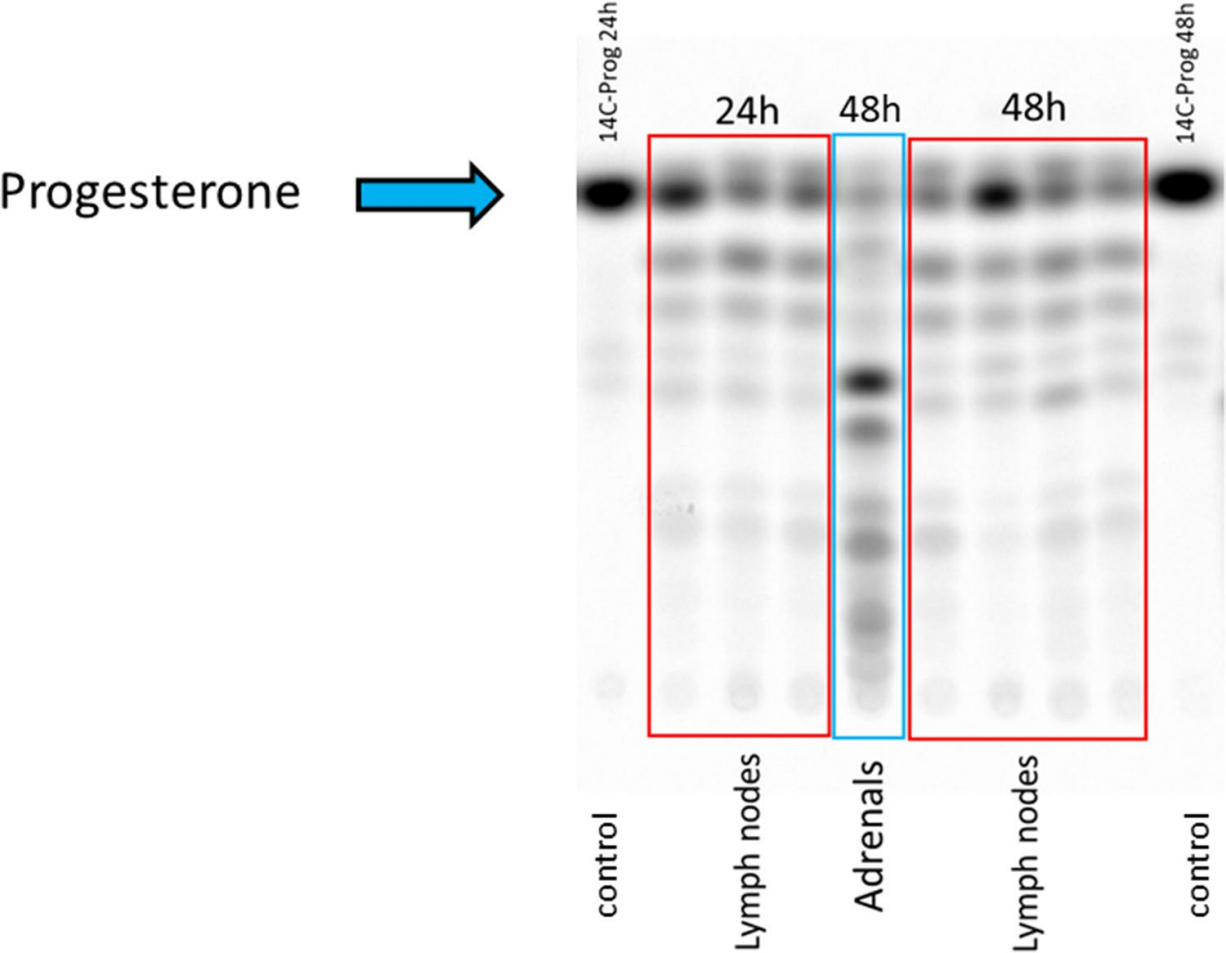
Progesterone metabolism in male mice lymph nodes and adrenal gland. Phosphorimager picture of TLC showing time-dependent conversion of progesterone to down-stream metabolites in lymph nodes. For positive control, adrenal glands are used under similar experimental conditions. Tissue-free controls were run for both time points. Line 1 at timepoint 24 h and line 2 at 48 h were entire lymph nodes, while all other lymph nodes were cut in half. The figure shows results of 7 isolated lymph nodes. Each lymph node was from a separate mouse. * p < 0.05, ** p < 0.01, *** p < 0.0001, ns = not significant.

Mouse lymph nodes, isolated from male mice (n = 7), were incubated for 24 h and 48 h with ^14^C-progesterone. Progesterone metabolism was analysed in supernatants as described. Adrenal gland was used as positive control. Progesterone was converted into 7 different metabolites in all lymph nodes. The progesterone metabolism pattern was different as compared to the one found in the adrenal gland, confirming progesterone metabolism and not de-novo steroid hormone production (Fig. 7).

All assessed steroid hormones, enzymes and steroidogenic factors in this manuscript are summarized in Supplementary Fig. 2 online in a schematic view.

### Immune response of CD4+ and CD8+ T cells upon stimulation with progesterone metabolites

**Figure 8:**
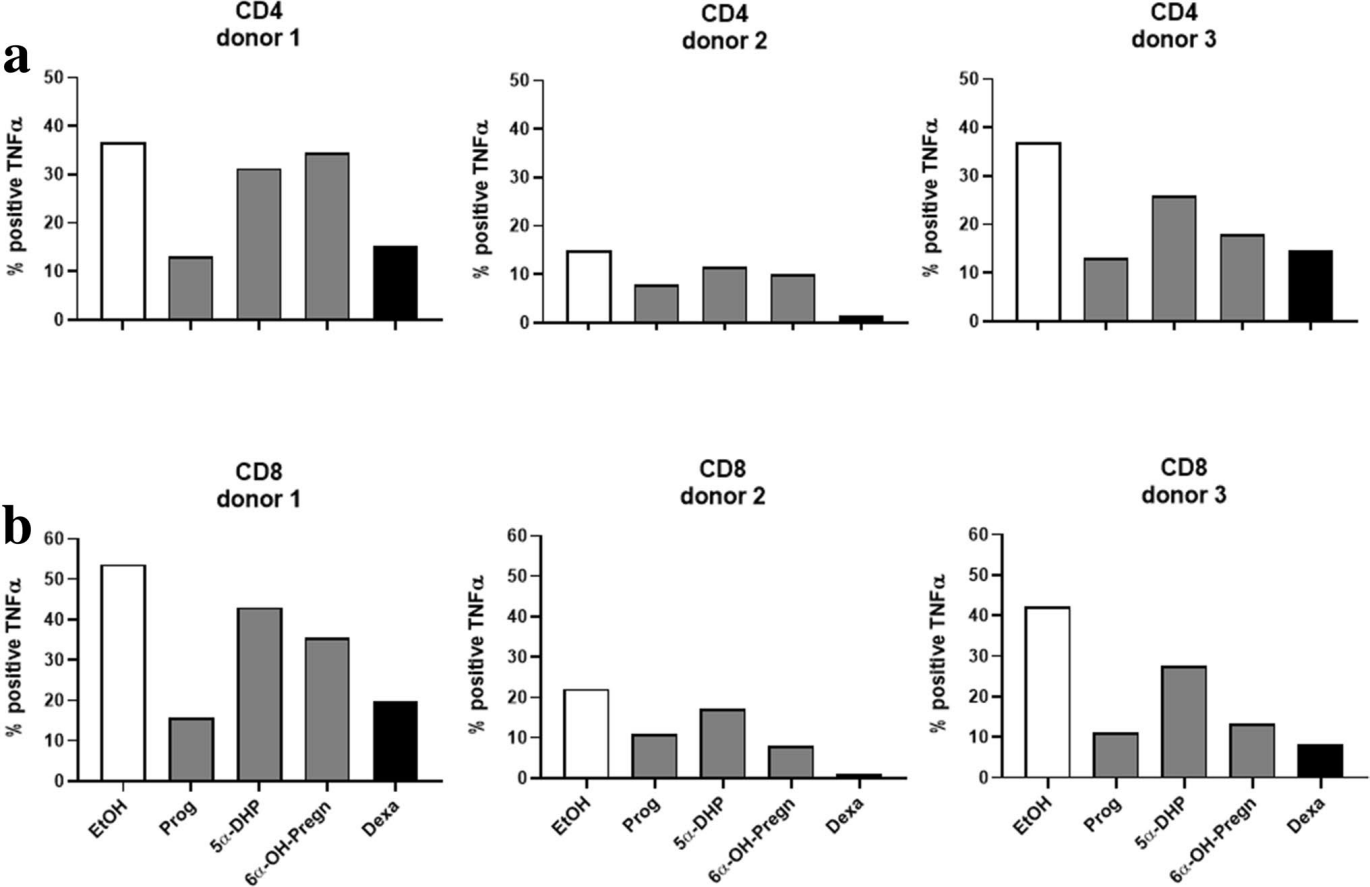
TNF-α production in CD4+ and CD8+ T cells upon stimulation with progesterone metabolites. Progesterone (Prog), 5α-dihydroprogesterone (5α-DHP), 6α-hydroxypregnanolone (6α-OH-Pregn) and dexamethasone (Dexa), were added at a concentration of 10^−3^ M to the PBMCs obtained from 3 different female donors for 24 h. 6 h after first steroid hormone contact, PBMCs were activated with PMA/Ionomycin. TNF-α positive CD4+ and CD8+ T cells were counted by FACS analysis. The y-axis shows the % of CD4+ (**a**) and CD8+ (**b**) T cells positively staining for TNF-α. * *p* < 0.05, ** *p* < 0.01, *** *p* < 0.0001, ns = not significant.

**Figure 9:**
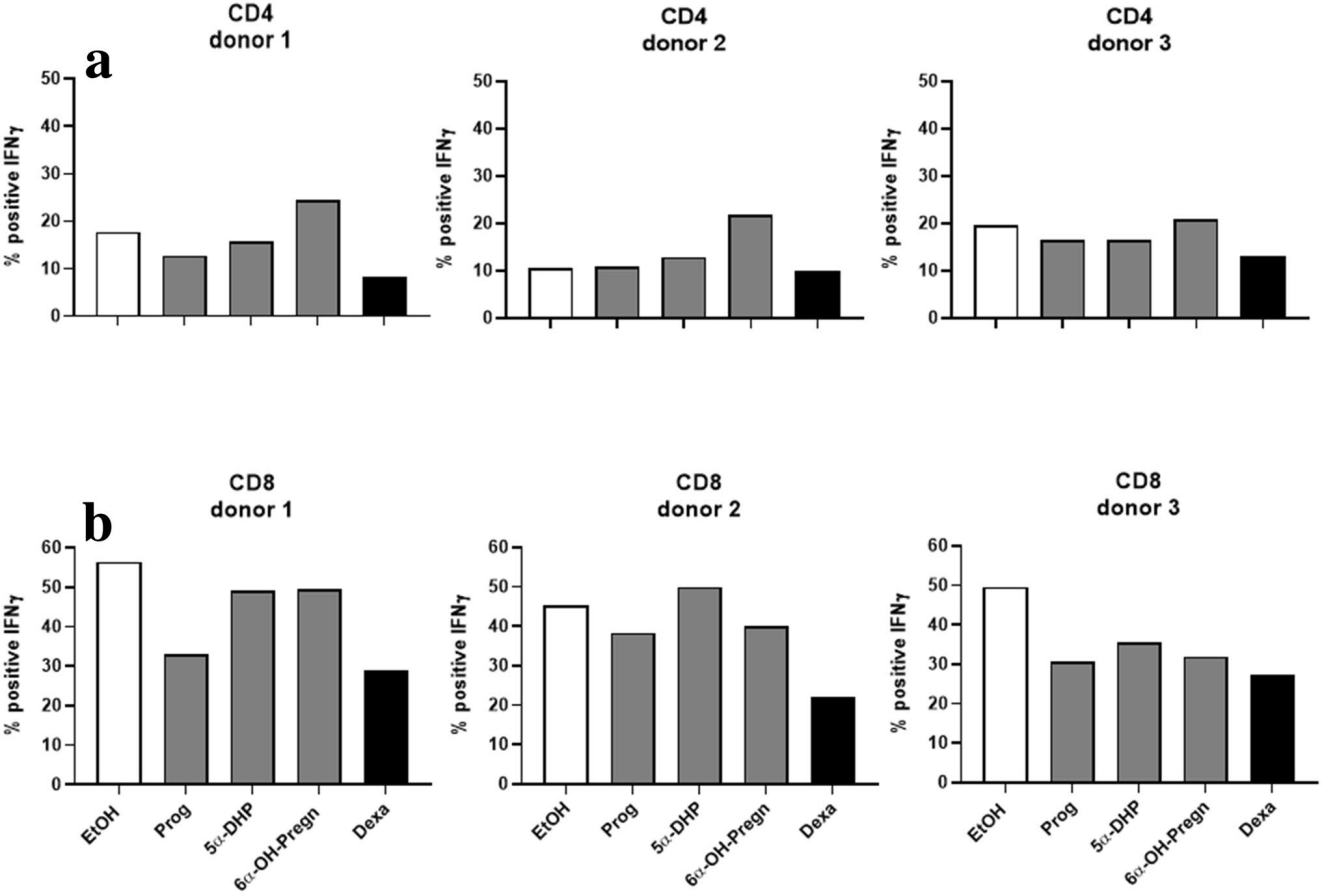
IFN-γ production in CD4+ and CD8+ T cells upon stimulation with progesterone metabolites. Progesterone (Prog), 5α-dihydroprogesterone (5α-DHP), 6α-hydroxypregnanolone (6α-OH-Pregn) and dexamethasone (Dexa), were added at a concentration of 10^−3^ M to the PBMCs of 3 different female donors for 24 h. 6 h after first steroid hormone contact, PBMCs were activated with PMA/Ionomycin. IFN-γ positive CD4+ and CD8+ T cells were counted by FACS analysis. The y-axis shows the % of CD4+ (**a**) and CD8+ (**b**) T cells positively staining for IFN-γ. * *p* < 0.05, ** *p* < 0.01, *** *p* < 0.0001, ns = not significant.

PBMCs of three different healthy female donors were isolated and pre-incubated with the steroid hormones progesterone, 5α-DHP, 6α-OH-pregnanolone, and with dexamethasone (Dexa) as positive control for 6 h before activation. Solvent control of all steroid hormones was EtOH. Activation of T cells was thereafter induced by PMA/Ionomycin. TNF-α and IFN-γ production was assessed 24 h later using FACS analysis. Progesterone and Dexa, strongly decreased TNF-α positive CD4+ (Fig. 8a) and CD8+ (Fig. 8b) T cell populations. 5α-DHP and 6α-OH-pregnanolone reduced the percentage of TNF-α positive CD4+ T cell subpopulations only in donor 3, while both progesterone metabolites reduced the percentage of TNF-α positive CD8+ T cells in all three donors (Fig. 8). Dexa strongly decreased IFN-γ positive CD4+ (Fig. 9a) and CD8+ (Fig. 9b) T cell populations. Progesterone reduced % of IFN-γ positive CD8+ T cells in all three donors, while it inhibited IFN-γ positive CD4+ T cells only in donor 1. The progesterone metabolites 5α-DHP and 6α-OH-pregnanolone showed a strong effect in CD8+ T cells of donor 3, where both steroids decreased IFN-γ positive CD8+ T cells by 20% (Fig. 9).

## Discussion

To our knowledge, this is the first report showing detailed steroid hormone metabolism in the lymphatic system. The lymphatic endothelium could be identified as an important regulator of steroid hormone bioavailability, yet also as a target of steroid hormones by expressing the respective steroid hormone receptors. *De-novo* steroid hormone synthesis from cholesterol is absent in lymphatic tissue, which is consistent with the lack of steroidogenic acute regulatory protein (StAR) in LECs. However, FDXR, FDX1 and LRH-1, genes involved in steroidogenesis^40–42^, are expressed in LECs. Progesterone, a steroid hormone critical in regulating immune responses and pregnancy, is intensely metabolized. In primary human LECs derived either from lymph nodes or from dermal lymphatic vessels, progesterone is mainly converted to a single metabolite, which was identified as 6α-OH-pregnanolone (5α-pregnan-3α, 6α-diol-20-one). Enzymatic steps involved are the two isoforms of the 5α-reductase, SRD5A1 and SRD5A3, the isoform 1 of 3α-HSD (AKR1C1) and a 6α-hydroxylase. Besides converting progesterone to the down-stream metabolites 5α-dihydroprogesterone (5α-DHP), allopregnanolone and 6α-OH-pregnanolone, HLECs and dLECs were able to reactivate progesterone from the less potent metabolite 20α-OHP. Further steroidogenic activity of HLEC and dLEC included deactivation of testosterone and 17β-estradiol to androstenedione and estrone as well as androstenediol to DHEA and DHEA-S.

These findings are pivotal in understanding the progesterone-dependent immunomodulation observed earlier by various groups^7,10,11,43^. The data show that progesterone is not only metabolized to the primary and active metabolite 5α-DHP which plays a critical role in pregnancy^44^, but is also restored from 20α-OHP.

Progesterone metabolism to 6α-OH-pregnanolone and the reactivation of progesterone from 20α-OHP by LECs serves to adapt immune tolerance and to control local and/or systemic progesterone bioavailability in high progesterone conditions as found during pregnancy. As the exact mechanism for this phenomenon is unknown and given the huge surface of the lymphatic endothelium throughout the body, it is supposable that local progesterone metabolism in LECs could be the key regulator in preventing cancer cells from being destroyed.

The direct immunosuppressive role of progesterone in both reproduction and tumor progression was impressively shown by Szekeres-Bartho and Polgar^45^. Immunosuppression by progesterone is mediated via the progesterone regulated gene PIBF1^8,11,13,46^, which has now also been found to be highly expressed in LECs. A reduced expression of PIBF in threatened pregnancies^47–51^ and an increased production of PIBF in cancer^52,53^ are clearly linked to a failed immune modulation. Production of TNF-α and IFN-γ in activated CD4+ and CD8+ T cells from 3 different donors was modulated upon stimulation with progesterone and its metabolites. It needs to be investigated if the interaction of LECs and the immune cells is PIBF1 regulated.

Steroid hormone effector mechanisms such as the glucocorticoid receptor (GR), the mineralocorticoid receptor (MR), the G protein-coupled estrogen receptor 1 (GPER1), and the progesterone receptor membrane components PGRMC1 and PGRMC2 are present in HLECs and dLECs, while no expression of nuclear progesterone receptors (PRA and PRB) was found.

Most of the known anti-inflammatory effects of progesterone are transmitted through the GR, with progesterone binding to the GR even though progesterone can also bind to PGRMCs^54,55^. PGRMCs were overexpressed in the maternal–fetal interface and in the embryonic/fetal trophectoderm in pregnancy^56,57^ as well as in T cells during pregnancy^58,59^. Since many steroid hormone receptors are expressed by immune cells^12,60–62^ it is most likely that the LECs not only have an autocrine, but also a paracrine role in controlling immune responses through the action of steroid hormones via binding to a receptor.

The serum concentrations of progesterone are according to the literature much too low to support the concept of a generalized immunosuppression^63,64^ and are only sufficient to inhibit peripheral natural killer cell activity in normal pregnant women. The presence of lymphatic vessels throughout the body, together with the immunosuppressive role of the placenta, might be a much better explanation for the systemic immunosuppression.

The mononuclear phagocyte system stimulates VEGF-C release and regulates thereby lymphangiogenesis^65^. High progesterone levels seem to coincide with high VEGF-C expression^66^. Activated macrophages and monocytes releasing VEGF-C thus enhance LEC growth and could well contribute to immune regulatory mechanisms via progesterone.

In 2009, Zhang et al. found, that in isolated mature adipocytes progesterone was converted to 20α-hydroxyprogesterone as the main metabolite, most likely through the activity of aldo–keto reductases AKR1C1, AKR1C2 and AKR1C3 (20α-HSD, 3α-HSD type 3 and HSD17B5), respectively^67^. Even though HLECs and dLECs express the same enzymes, 6α-OH-pregnanolone was found instead as major metabolite of progesterone, and not 20α-hydroxyprogesterone. That LECs favour the 5α-reductase/3α-HSD pathway over the 20α-HSD pathway might be explained by the different tissues investigated and eventually the limitations of some cofactors.

The strength of this study is the thorough analysis of different steroid hormone metabolites using state-of-the-art technology. It shows enzyme expression on mRNA and on protein level, as well as functional assays including inhibitory experiments. All results are referred to our self-designed and validated model of a positive control, the placental cell line HTR-8/SV neo. Since fast, significant and similar progesterone metabolism was found in HTR-8/SV neo cells, they were used as a model of reference for all experiments. Taking a placental cell line as model is obvious, as the placenta is the primary organ exposed to high progesterone levels. A direct immunologic effect of the lymphatic progesterone metabolites on CD4+ and CD8+ T cells could be shown as well.

Investigation of further immunomodulatory effects of the lymphatic progesterone metabolism and progesterone reactivation described herein on immune and cancer cells is the goal of future studies.

Verification of our observations in an in vivo model is difficult due to the systemic presence of steroid hormones, further complicated by the delicate structure of lymphatic vessels, therefore precluding differential concentration measurements. But, the capacity of steroid hormone metabolism and reactivation in lymph nodes isolated from both sexes, including pregnancy, should be explored. Additionally, future detailed assessment in patients treated with the 5α-reductase inhibitors dutasteride or finasteride could be investigated with more accuracy.

In conclusion, we identified and characterized the lymphatic system as major steroid hormone metabolizing tissue, which is additionally able to reactivate progesterone from 20α-OHP and therefore adapts progesterone availability. This is important in high progesterone conditions such as in pregnancy in order to adjust local immune tolerance. The impact of these findings on cancer and pregnancy research might be meaningful and could provide new targets for the treatment of cancer and pregnancy related diseases. Furthermore, these results could also help to elucidate disease mechanisms in autoimmunity and allergy.

## Methods

### Material and cell lines

Collagen I coated petridishes were from Corning (Milian, Nesselnbach, Switzerland). The primary HLEC (Cat. No. 2500, Lots. 19,399, 19,394 and 19,415) were from Sciencell (Chemie Brunschwig, Basel, Switzerland) and the primary human dLEC (CC-2810, Lots. 4F3029 and 4F3037) were from Lonza (Ruwag, Bettlach, Switzerland) or from PromoCell Heidelberg, Germany (C-12217, Lot 431Z021.3). Primary cells were obtained from female donors. The HTR-8/SVneo cells were a gift from Charles H. Graham (Queen’s University, Kingston, Ontario, Canada). The vascular cell basal medium (PCS-100-030) and the supplementary factors (PCS-100-041) were from ATCC (LGC Standards GmbH, Wesel, Germany). The Endothelial Cell Growth Medium EGM-2 (CC-3162), including EBM-2 Basal Medium (CC-3156) and EGM-2 Supplements (CC-4176), was from Lonza (Ruwag, Bettlach, Switzerland). The PromoCell MV-2 medium with supplements (C-22121) was from PromoCell (Heidelberg, Germany). Medium RPMI 1640 (Cat.Nr. 21,875) was from Gibco (ThermoFisher Scientific, Reinach, Switzerland). Penicillin/streptomycin (P/S, Cat. No. 0503) and FBS (Cat. No. 0025) were from Sciencell (Chemie Brunschwig, Basel, Switzerland). ^3^H-cholesterol (ART-0255), ^3^H-progesterone (ART-0795) and ^14^C-progesterone (ARC-1398) were from American radiolabeled chemicals (ARC) Inc. (Anawa Trading SA, Wangen ZH, Switzerland). Scintillation solution Optiphase Supermix (Cat. No. 1200-439) was from Perkin Elmer Schweiz AG (Schwerzenbach, Schweiz). The SRD5A-inhibitor dutasteride (SML 1221) was purchased from Sigma-Aldrich (Buchs, Switzerland).

Cholesterol, progesterone, and all steroid hormone standards for LC–MS and TLC analysis were purchased from Steraloids, Inc. (Newport, RI, USA). Pre-coated TLC plates SIL G25 UV_254_ (REF 809,023) and pre-coated TLC sheets Polygram SIL G/UV_254_ (REF 805,023) were from Macherey–Nagel GmbH, Düren, Germany. Dichloromethane (1.06050), methanol (1.06009) and choloroform (1.02445) were from Merck Millipore, Zug, Switzerland. Microbeta 2 Microplate counter 2450 was from Perkin Elmer Schweiz AG (Schwerzenbach, Schweiz). Phosphorimager Typhoon FLA 7000 and the Storage Phosphor Screen (BAS-IP TR 2040 E Tritium Screen) were from GE Healthcare Life Sciences, Glattbrugg, Switzerland. Prime Script RT Reagent Kit Cat. RR037A was from Takara Bio Europe (Saint-Germain-en-Laye, France).

For LC–MS analysis, a Vanquish UHPLC (equipped with an ACQUITY UPLC HSS T3 Column, 100 Å, 1.8 µm, 1 mm X 100 mm column; Waters, Switzerland) was coupled to a Q Exactive Plus Orbitrap (both Thermo Fisher Scientific, Reinach, Switzerland). Separation was achieved using gradient elution over 11 min using water and methanol both supplemented with 0.1% formic acid (all Sigma-Aldrich, Buchs, Switzerland) as mobile phases. Data analysis was performed using TraceFinder 4.1 (Thermo Fisher Scientific, Reinach, Switzerland). All steroids analyzed by LC–MS and their systematic names are listed in Table 7.

**Table 7:** List of all steroids analyzed or mentioned in this study with their short and systematic name.

Class	Short name	Systematic name
Progesterones	Pregnenolone	5-Pregnen-3β-ol-20-one
	Progesterone	4-Pregnen-3, 20-dione
	17OH-Progesterone	4-Pregnen-17-ol-3, 20-dione
	20α-Hydroxyprogesterone	4-Pregnen-20α-ol-3-one
	5α-Dihydroprogesterone	5α-Pregnan-3, 20-dione
	5β-Dihydroprogesterone	5β-Pregnan-3, 20-dione
	Pregnanolone	5β-Pregnan-3α-ol-20-one
	Epipregnanolone	5β-Pregnan-3β-ol-20-one
	Allopregnanolone	5α-Pregnan-3α-ol-20-one
	Isopregnanolone	5α-Pregnan-3β-ol-20-one
	6α-OH-Pregnanolone	5α-Pregnan-3α, 6α-diol-20-one
	6α-OH-Epipregnanolone	5α-Pregnan-3β,6α-diol-20-one
	Allopregnane-3,20-diol	5α-pregnan-3β,20α-diol
Mineralocorticoids	11-deoxycorticosterone	4-Pregnen-21-ol-3, 20-dione
	Corticosterone	4-Pregnen-11β, 21-diol-3, 20-dione
	Aldosterone	4-Pregnen-11β, 21-diol-3, 18, 20-trione
Estrogens	17β-Estradiol	1,3,5(10)-estratriene-3,17β-diol
	Estriol	1,3,5(10)-estratriene-3,16α,17β-triol
Glucocorticoids	11-deoxycortisol	4-Pregnen-17α,21-diol-3,20-dione
	Cortisone	17,21-dihydroxy-4-pregnene-3,11,20-trione
	Cortisol	11β,17,21-trihydroxy-4-pregnene-3,20-dione
Androgens	Androsterone	3α-hydroxy-5α-androstan-17-one
	Etiocholanolone	3α-Hydroxy-5β-androstan-17-one
	Dehydroepiandrosterone	5-androsten-3β-ol-17-one
	Dehydroepiandrosterone-sulphate	5-androsten-3β-ol-17-one sulphate
	5α-Dihydrotestosterone	17β-hydroxy-5α-androstan-3-one
	Testosterone	17β-hydroxy-4-androsten-3-one
	Androstenedione	4-androsten-3, 17-dione

Primer pairs for SRD5A1, SRD5A2, SRD5A3, AKR1D1, AKR1C1, AKR1C2, AKR1C3, AKR1C4, HSD3B1, HSD3B2, HSD17B2, HSD11B1, FDXR, and NR5A2 (Table 1) were designed by means of the Universal ProbeLibrary Assay Design Center software from Roche. All primers were intron-spanning and were synthesized by Microsynth AG (Balgach, Switzerland). Universal ProbeLibrary hydrolysis probes were purchased from Sigma-Aldrich (Buchs, Switzerland). Assay on demand primers for Cyclophilin A (4326316E), PIBF1, NR3C1, NR3C2, NR3C3, PGRMC1, PGRMC2, GPER1, CYP17A1, CYP21A2, CYP11B1, CYP11B2, HSD11B2, StAR, FDX1 (Table 2), and the 7500 Fast Real-time PCR system machine were from Applied Biosystems (Thermo Fisher Scientific, Reinach, Switzerland).

The MS part was done by the Proteomic Mass Spectrometry Core Facility PMSCF at DBMR in Bern, Switzerland.

For FACS analysis, GolgiPlug, anti-CD3 V500 (clone SK7), anti-CD4 PerCP (clone SK3) and anti-CD8 PE-Cy7 (clone SK1) were purchased from BD Biosciences. The fixable Live/Dead dye was obtained from Invitrogen. Anti-TNF-α AF647 (clone Mab11) and anti-IFN-γ BV421 (clone 4sB3) and their matched isotype controls were bought from Biolegend. PMA/Ionomycin was obtained from Sigma Aldrich.

All methods used in this manuscript were carried out in accordance with relevant guidelines and regulations.

### Cell culture

Primary HLECs and dLECs were cultured in collagen I coated cell ware. Medium for HLECs was the vascular cell basal medium from ATCC with all supplementary factors but without cortisol (hydrocortisone). FBS concentration was 5%. Medium for dLECs was the EBM-2 Basal Medium from Lonza with all EGM-2 supplements but without cortisol. FBS concentration was 2%. Primary HLECs and dLECs were used up to passage 6.

HTR-8/SV neo cells were cultured in RPMI 1640 5% FBS.

In order to minimize steroid hormone contamination in our medium or FBS, all experiments were performed in cortisol-free medium containing 5% or 2% of charcoal treated (ct) FBS.

PBMCs were cultured in RPMI1640 containing 10% FBS after isolation. During experiments, PBMCs were maintained in steroid-free RPMI1640.

### Thin layer chromatography

#### ^14^C-progesterone and ^3^H-cholesterol as substrate for HLEC and dLEC

HLECs and dLECs were cultured in collagen I coated 6-well plates with medium containing 5% or 2% ct FBS for 24 h. After 24 h cells were washed twice with DPBS and 0.05 μCi ^14^C-progesterone or 1 μCi ^3^H-cholesterol were added in steroid free medium. Cell culture supernatant was collected into glass vials at the indicated time points. 5 ml ethyl acetate was added and glass vials were vortexed for exactly 1 min. Thereafter, supernatants were stored at − 20 °C until phase separation. The upper phase was transferred into new glass vials and evaporated under a nitrogen stream at 56 °C. The pellet was suspended in 30ul of cold progesterone dissolved in EtOH (10 mg/ml) for the ^14^C-experiments and in 30ul of the cold mixture cholesterol, progesterone, corticosterone, cortisol, testosterone and estradiol (all 10 mg/ml EtOH) for the ^3^H-cholesterol experiments in HLEC. For the ^3^H-cholesterol experiments in dLEC, the pellet was dissolved in 30ul of the cold mixture of cholesterol, progesterone, 11-deoxycortisol, corticosterone, aldosterone and cortisol (all 10 mg/ml EtOH). A 10ul aliquot of each sample was loaded on TLC glass plates or sheets. Running medium was dichloromethane, methanol, H_2_O (150:10:1). For Microbeta 2 measurements, spots were visualized and marked under the UV lamp. Marked spots were scratched into scintillation vials. 3.5 ml of scintillation fluid was added and vials were counted in a Microbeta 2. Individual steroid hormones were run on the same plate to allow for localization of the steroid hormones. A control without cells (0 h/48 h for HLEC and dLEC; 0 h/24 h for HTR-8/SV neo) was loaded as baseline/background. Counts are displayed in counts per minute (CPM = CCPM1). Conversion of ^14^C-progesterone to 6α-OH-pregnanolone or of ^3^H-cholesterol to progesterone, corticosterone, cortisol, testosterone and estradiol (HLEC), or of ^3^H-cholesterol to progesterone, 11-deoxycortisol, corticosterone, aldosterone and cortisol (dLEC), was calculated. For visualization by a phorphorimager, TLC sheets were exposed to a Storage Phosphor Screen. Spots were visualized by the Phosphorimager Typhoon FLA 7000. Individual steroid hormones were run on the same sheet to allow for localization of the steroid hormones. A control without cells (0 h/48 h for HLEC and dLEC; 0 h/24 h for HTR-8/SV neo) was loaded as baseline/background. Quantification was done using ImageJ software. All conversion rates were compared to the controls without cells. Progesterone substrate availability at timepoint 0 h (control without cells) was taken as 100% for all experiments.

#### SRD5A-inhibition with dutasteride

HLECs and dLECs were cultured as described above and incubated with dutasteride dissolved in DMSO and used at a final concentration of 10^−5^ M and 10^−6^ M for HLECs and HTR-8/SV neo and 10^−6^ M and 10^−8^ M for dLECs. Total incubation time was 24 h. Conversion of progesterone to 6a-OH-pregnanolone was quantified by TLC.

#### ^*14*^*C-progesterone as substrate for HTR-8/SV neo*

HTR-8/SV neo were cultured in RPMI 1640 medium containing 5% ct FBS for 24 h. After 24 h cells were washed twice with DPBS and 0.05 μCi ^14^C-progesterone was added in steroid free medium. Cell culture supernatant was collected into glass vials at timepoints 1 h, 4 h, 8 h and 24 h. 5 ml ethyl acetate was added and glass vials were vortexed for exactly 1 h. The rest of the procedure was done exactly as described above for the HLEC and dLEC.

### Real-time PCR

mRNA expression of the progesterone metabolizing enzymes (*SRD5A1, SRD5A2, SRD5A3, AKR1D1, AKR1C1, AKR1C2, AKR1C3, AKR1C4, HSD3B1, HSD3B2*), of *PIBF1*, of the steroid hormone receptors (*NR3C1, NR3C2, NR3C3, PGRMC1, PGRMC2, GPER1*) of the steroidogenic enzymes (*CYP17A1, CYP21A2, CYP11B1, CYP11B2, HSD11B1, HSD11B2, HSD17B2*) and of the steroidogenic proteins *StAR, FDXR, FDX1, NR5A2 in HLEC, dLEC* and *HTR-8/SV* neo.

HLECs and dLECs were cultured in collagen I coated 6-well plates with medium containing 5% or 2% FBS for 24 h. HTR-8/SV neo were cultured in RPMI 1640 medium containing 5% FBS for 24 h. After the incubation time total extraction of RNA was performed using the Trizol method. RNA was reverse transcribed using the Prime Script RT Reagent kit from Takara. Real-time PCR was performed using assay on demand primers or primers and probes designed with the Roche Library in order to detect the following genes: *SRD5A1, SRD5A2, SRD5A3, AKR1D1, AKR1C1, AKR1C2, AKR1C3, AKR1C4, HSD3B1, HSD3B2, PIBF1, NR3C1, NR3C2, NR3C3, PGRMC1, PGRMC2, GPER1, CYP17A1, CYP21A2, CYP11B1, CYP11B2, HSD11B1, HSD11B2, HSD17B2, StAR, FDXR, FDX1*, and *NR5A2* (Tables 1, 2). Cyclophilin A served as endogenous control. Assays were performed in duplicates.

### Proteomics

HLEC and dLEC were cultured in their medium containing 5% or 2% FBS. Upon confluency, cells were washed 2 × with DPBS, detached with trypsine/EDTA (1 × concentrated) and centrifuged at 800 rpm. Cell pellet was washed 2 × with DPBS and the dried pellet was lysed in 8 M UREA buffer containing a protease inhibitor cocktail. Protein amount was measured by the Qubit Protein Assay. The MS part was done by the Proteomic Mass Spectrometry Core Facility PMSCF using standard procedure.

### LC–MS

HLECs and dLECs were cultured in collagen I coated 6-well plates with medium containing 5% ct FBS for 24 h. After 24 h cells were washed twice with DPBS and the cold testosterone, 17β-estradiol, 5α-dihydroprogesterone and androstenediol were added separately in steroid-free medium at a concentration of 10^−6^ M. A time-course experiment was performed with incubation times 0 h as baseline, 4 h and 24 h for HLEC and 0 h and 24 h for dLEC. Steroids were extracted from the supernatant by solid phase extraction using Oasis HLB SPE plates (Waters, Switzerland). Remaining substrate concentrations and metabolites were assessed using LC–MS. 20 uL of the extract were injected and separation was achieved using gradient elution over 11 min using water and methanol both supplemented with 0.1% formic acid as mobile phases. 6α-OH-pregnanolone was identified based on accurate mass measurement, comparison of MS/MS spectra and retention times between the analyte in the samples and an authentic standard. Data was normalized to condition at 0 h = 1000 nM = 10^−6^ M for each compound.

### Mouse lymph nodes and adrenals

Animal experimentation was approved by the Ethics Committee for Animal Experiments of the Veterinary Administration of the Canton of Berne, Switzerland and conformed to the rules of the Swiss Federal Act on Animal Protection (BE58/19). C57/Bl6 mice were bred according to the rules in the central animal facility of the university of Bern. They were maintained under 12-h dark–light cycles with unrestricted access to food and water. Males used in experiment were fed for 6–8 weeks with regular chow diet. On the day of sacrifice, lymph nodes and adrenal glands were isolated and put on ice until arrival at the lab. Thereafter, organs were washed in PBS and put into warm, steroid-free medium (PCS-100-030) containing 5% ct FBS. The fat was dissected, and the adrenal gland, and half of the lymph nodes were cut in half. The rest of the lymph nodes were uncut and contained a capsule. Every lymph node (cut or entirely), and the adrenal gland were placed in 48-well plates and incubated with 500ul of steroid-free medium containing 5% ct FBS and 0.05 µCi ^14^C-progesterone for 24 h or 48 h at 37 °C. For controls, medium from wells incubated under the same conditions and incubation time but without tissue was used. After 24 h and 48 h supernatants were collected into glass vials. 5 ml ethyl acetate was added and vials were vortexed exactly for 1 min. The next steps were performed as described in the section thin layer chromatography. Products of progesterone metabolism were visualized with a Phosphorimager.

### FACS

Peripheral blood mononuclear cells (PBMCs) were isolated from three healthy, non-pregnant female donors using Ficoll gradient centrifugation. After isolation, PBMCs were kept for 30 min in RPMI1640 medium containing 10 mM HEPES, 10% heat-inactivated FBS and 100 μg/ml Streptomycin, 100U/ml penicillin (all from Gibco, Life Technologies). After 30 min, PBMCs were stimulated with progesterone, 5α-DHP, 6α-OH-pregnanolone and dexamethasone for 6 h (priming). Control medium was RPMI1640 containing EtOH, the solvent of the steroid hormones. Final concentration of all steroid hormones on PBMCs was 10^−3^ M. After the 6 h pre-incubation period with steroid hormones, lymphocytes were activated with 15 ng/ml PMA and 1 μg/ml Ionomycin. Simultaneously, GolgiPlug was added in order to inhibit cytokine release. After a total incubation period of 24 h, PBMCs were stained for CD3, CD4, CD8, IFN-γ and TNF-α and analyzed by FACS as follows: Cells were stained with fixable Live/Dead dye for 30 min one ice. Subsequently cells were washed using PBS and fixed by adding Medium A from the Fix & Perm kit (Invitrogen) for 15 min at RT. Afterwards cells were washed and the permeabilization Medium B and the antibody cocktail containing anti-CD3, anti-CD4, anti-CD8, anti-IFN-γ and anti-TNF-α was added. Cells were incubated for 20 min, washed and resuspended in PBS containing 2% heat-inactivated FBS, 0.05% sodium azide. Data was acquired using a FACSCanto II (BD Biosciences). Matched isotype controls were used for anti-IFN-γ and anti-TNF-α Percentage of IFN-γ and TNFα positive cells was determined in alive cells, which were CD3 positive.

The % of CD4+ and CD8+ T cells owning the intracellular cytokines IFN-γ and TNF-α is shown in Fig. 8 (TNF-α) and Fig. 9 (IFN-γ).

### Statistics

All experiments were performed at least 3 times. Pictures in figures are unprocessed. Representative blots are shown. Densitometry was performed for all phosphorimager pictures using Image J software. Conversion and production rates are displayed as mean ± SD using one-way ANOVA with Dunnetts multiple comparison test. Significance was assigned at *p* < 0.05. TLC experiments were additionally controlled by CCPM1 measurements in a microbeta2 instrument. For TaqMan results ct values were calculated as mean of all experiments. All statistical analyses were performed using GraphPad PRISM version 8 (PRISM, USA).

#### Equipment and settings

For the phosphorimager pictures in Figs. 1, 5 and 7 the image acquisition tool Image J was used. No manipulations have been made to the pictures.

## Supplementary Information

Supplementary Information

## Data Availability

All data generated or analysed during this study are included in this published article (and its supplementary information files online).
