# Supplementary material for: Steroid hormone bioavailability is controlled by the lymphatic system
Source: Sci Rep. 2021 May 6;11:9666. doi: 10.1038/s41598-021-88508-w (PMC8102502; doi:10.1038/s41598-021-88508-w)
Supplement: Supplementary file 1 — Supplementary Information [file 41598_2021_88508_MOESM1_ESM.pptx]

## Slide 1
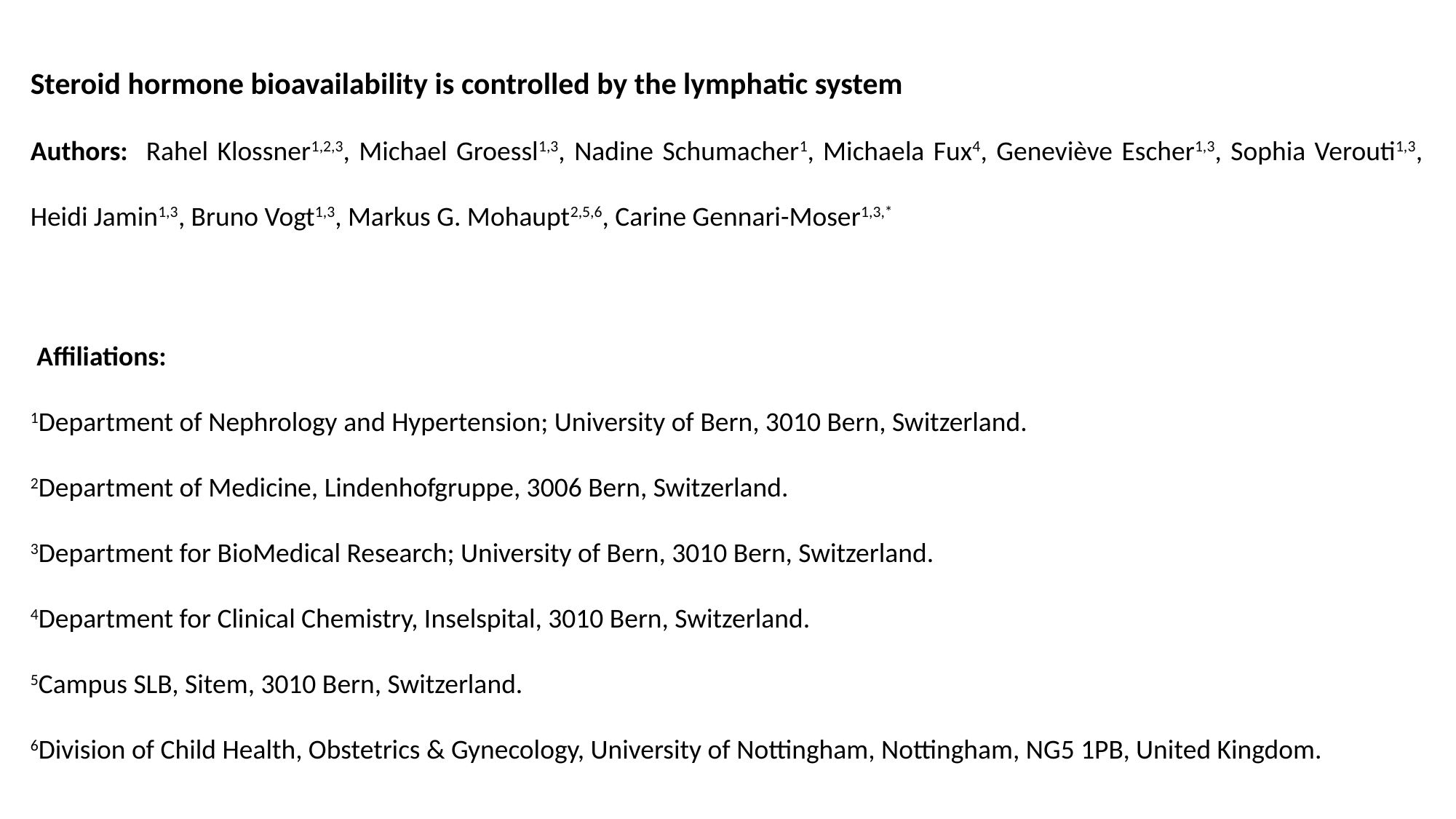

Steroid hormone bioavailability is controlled by the lymphatic system
Authors: Rahel Klossner1,2,3, Michael Groessl1,3, Nadine Schumacher1, Michaela Fux4, Geneviève Escher1,3, Sophia Verouti1,3, Heidi Jamin1,3, Bruno Vogt1,3, Markus G. Mohaupt2,5,6, Carine Gennari-Moser1,3,*
 Affiliations:
1Department of Nephrology and Hypertension; University of Bern, 3010 Bern, Switzerland.
2Department of Medicine, Lindenhofgruppe, 3006 Bern, Switzerland.
3Department for BioMedical Research; University of Bern, 3010 Bern, Switzerland.
4Department for Clinical Chemistry, Inselspital, 3010 Bern, Switzerland.
5Campus SLB, Sitem, 3010 Bern, Switzerland.
6Division of Child Health, Obstetrics & Gynecology, University of Nottingham, Nottingham, NG5 1PB, United Kingdom.

## Slide 2
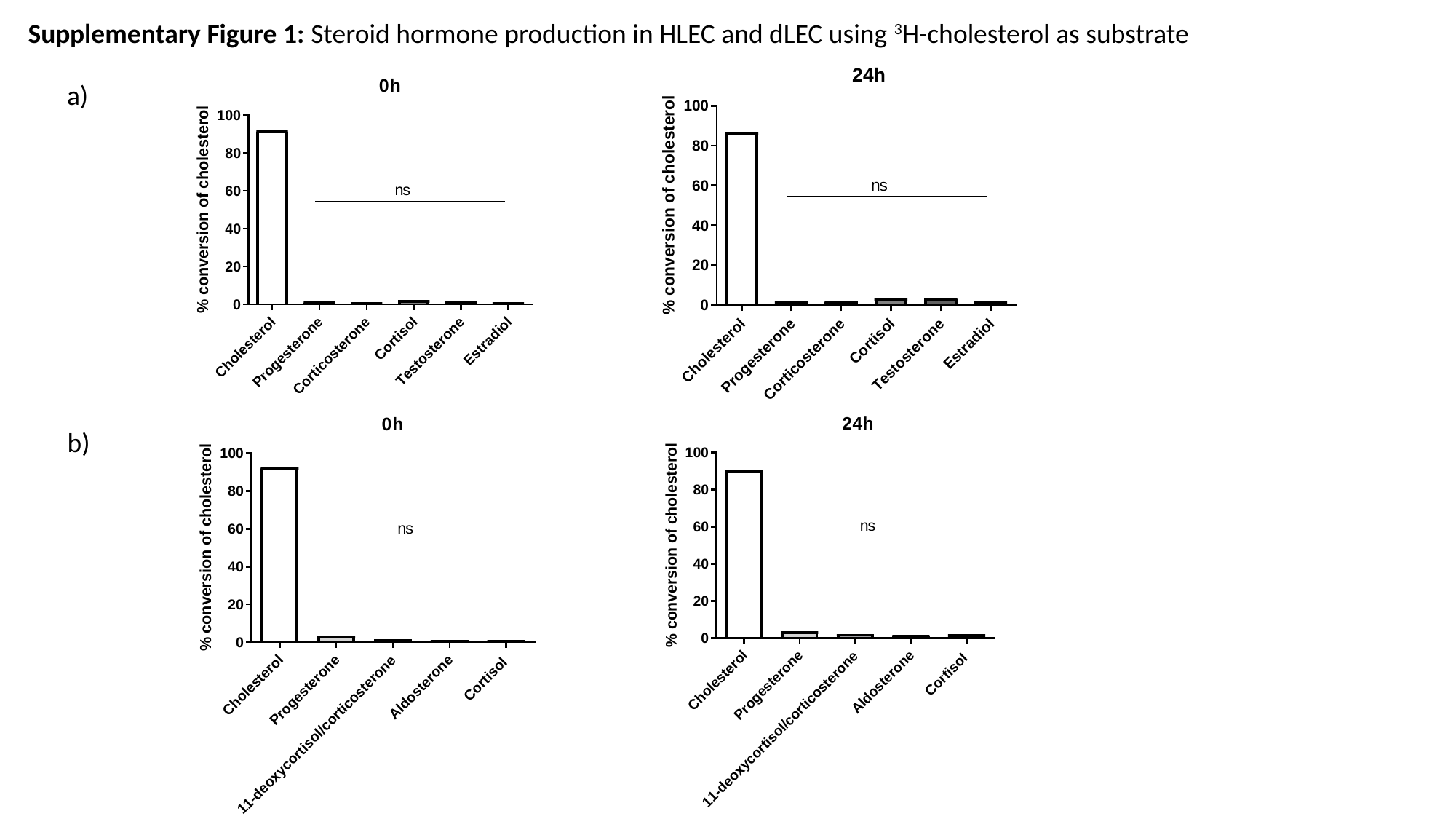

Supplementary Figure 1: Steroid hormone production in HLEC and dLEC using 3H-cholesterol as substrate
a)
b)

## Slide 3
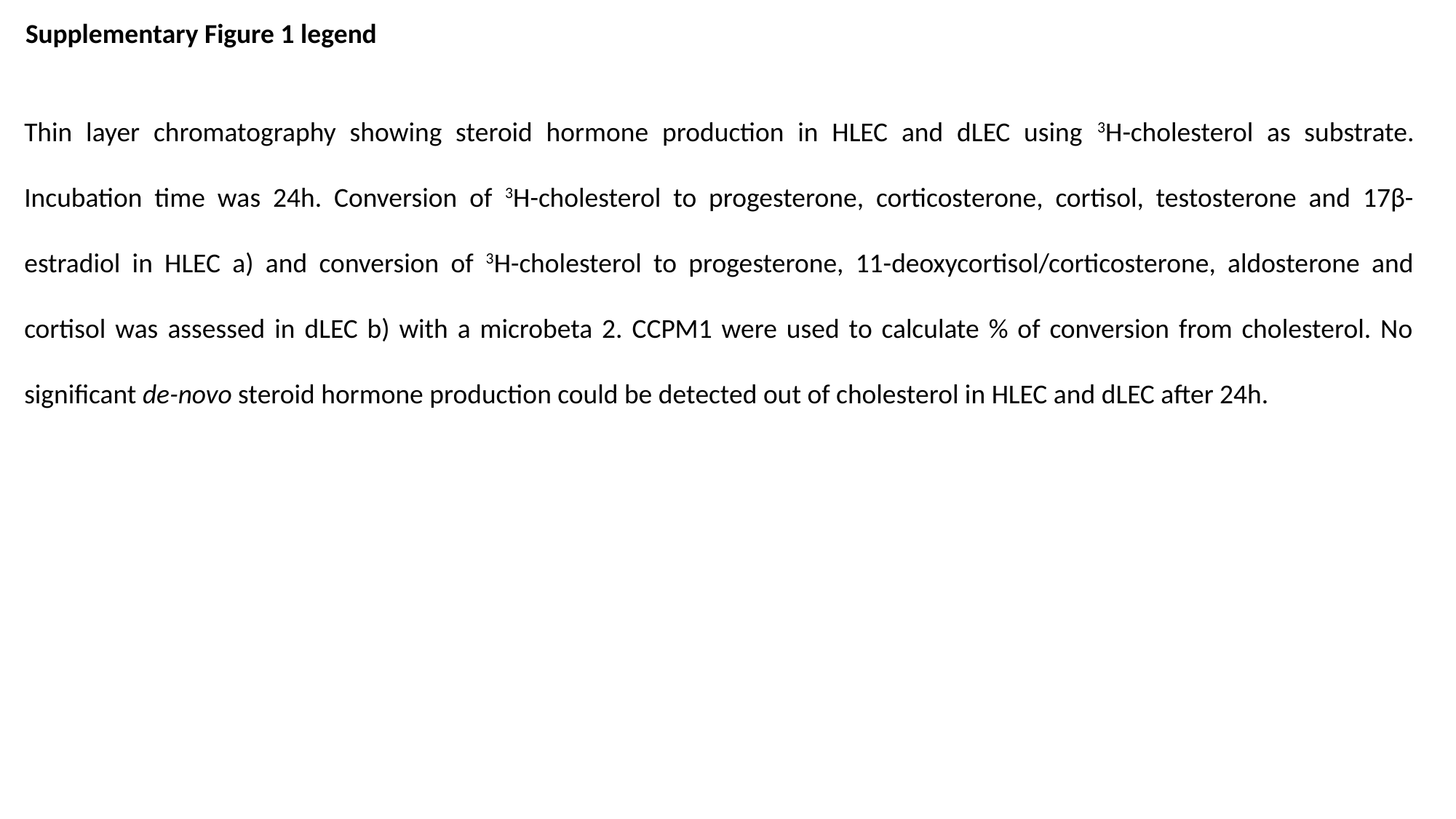

Supplementary Figure 1 legend
Thin layer chromatography showing steroid hormone production in HLEC and dLEC using 3H-cholesterol as substrate. Incubation time was 24h. Conversion of 3H-cholesterol to progesterone, corticosterone, cortisol, testosterone and 17β-estradiol in HLEC a) and conversion of 3H-cholesterol to progesterone, 11-deoxycortisol/corticosterone, aldosterone and cortisol was assessed in dLEC b) with a microbeta 2. CCPM1 were used to calculate % of conversion from cholesterol. No significant de-novo steroid hormone production could be detected out of cholesterol in HLEC and dLEC after 24h.

## Slide 4
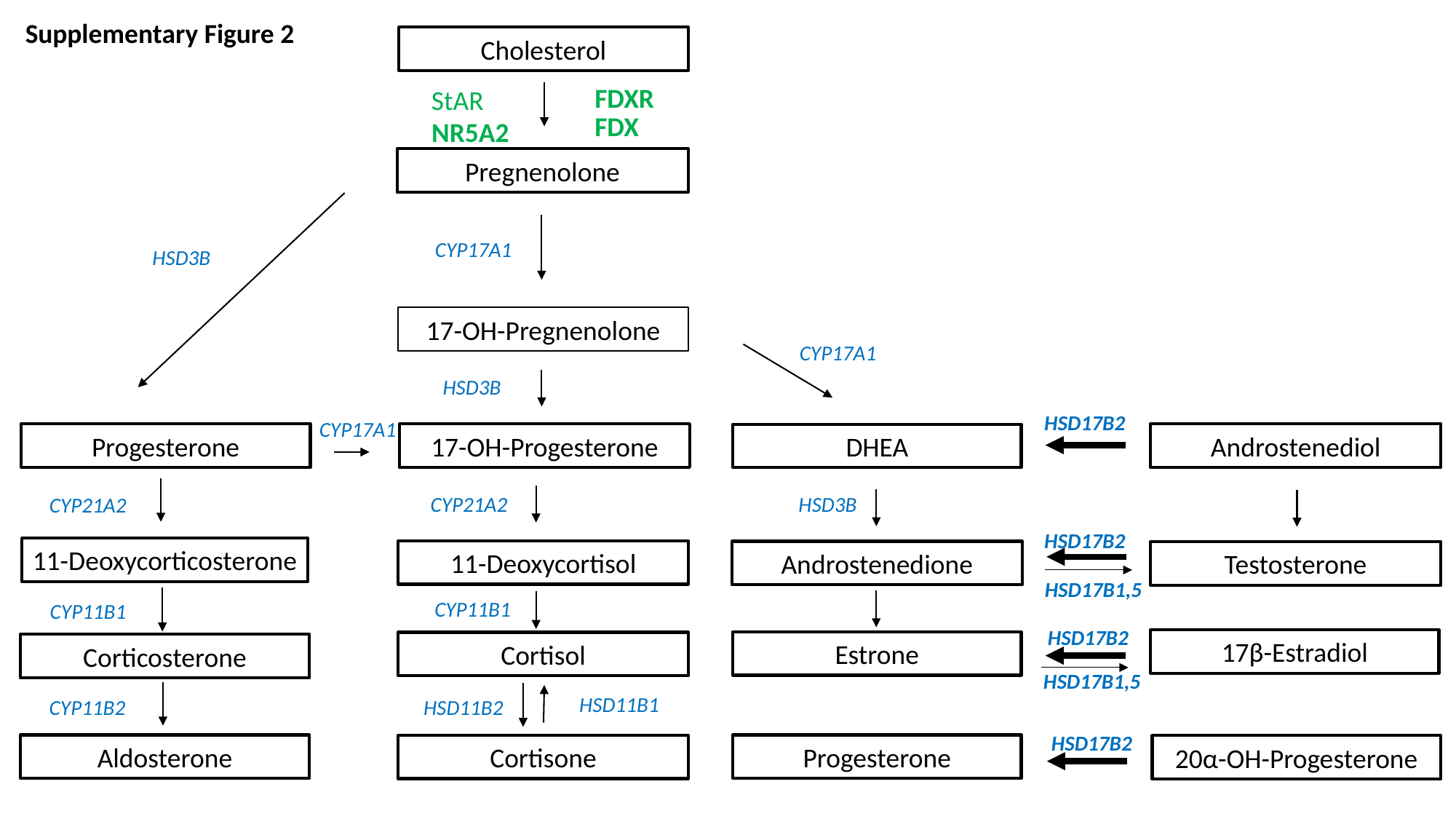

Supplementary Figure 2
Cholesterol
FDXR
StAR
FDX
NR5A2
Pregnenolone
CYP17A1
HSD3B
17-OH-Pregnenolone
11-Deoxycortisol
Cortisol
Cortisone
CYP17A1
HSD3B
HSD17B2
CYP17A1
Progesterone
17-OH-Progesterone
Androstenediol
DHEA
CYP21A2
HSD3B
CYP21A2
HSD17B2
11-Deoxycorticosterone
Androstenedione
Testosterone
HSD17B1,5
CYP11B1
CYP11B1
HSD17B2
17β-Estradiol
Estrone
Corticosterone
HSD17B1,5
HSD11B1
HSD11B2
CYP11B2
HSD17B2
Aldosterone
Progesterone
20α-OH-Progesterone

## Slide 5
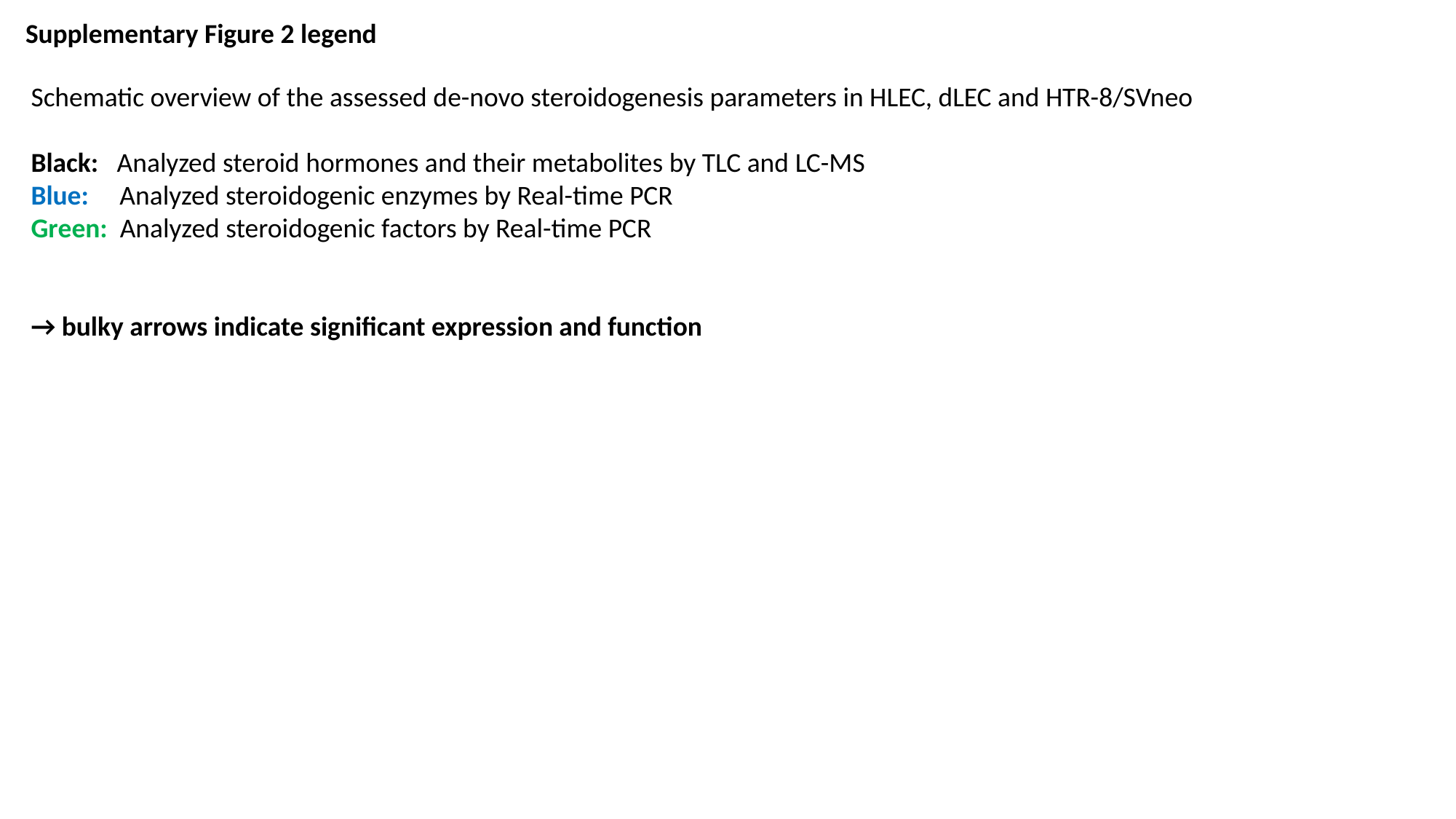

Supplementary Figure 2 legend
Schematic overview of the assessed de-novo steroidogenesis parameters in HLEC, dLEC and HTR-8/SVneo
Black: Analyzed steroid hormones and their metabolites by TLC and LC-MS
Blue: Analyzed steroidogenic enzymes by Real-time PCR
Green: Analyzed steroidogenic factors by Real-time PCR
→ bulky arrows indicate significant expression and function
